# *N*-Glycosides of indigo, indirubin, and isoindigo: blue, red, and yellow sugars and their cancerostatic activity

**DOI:** 10.3762/bjoc.20.240

**Published:** 2024-11-08

**Authors:** Peter Langer

**Affiliations:** 1 Institut für Chemie, Universität Rostock, Albert-Einstein-Str. 3a, 18059 Rostock, Germany

**Keywords:** cancerostatic activity, carbohydrates, heterocycles, *N*-glycosides, indirubin

## Abstract

Indigo, indirubin, and isoindigo derivatives have been used for centuries as pigments. Since the 1990s, a new aspect of the chemistry of this type of compounds is their activity against various types of cancer. *N*-Glycosides of indigo, indirubin, and isoindigo, blue, red, and yellow sugars, turned out to be of special interest because of their high cancerostatic activity and structural novelty. The present article provides an account on the synthesis and anticancer activity of these compounds.

## Introduction

**Scheme 1:**
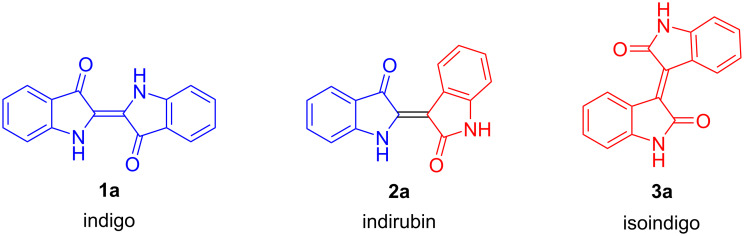
Structures of indigo (**1a**), indirubin (**2a**) and isoindigo (**3a**).

Indigo (**1a**), known for more than 6000 years and originally produced from indigo plants in India, represents a famous traditional blue pigment which was an expensive material in Europe (Scheme 1). It is obtained by extraction and isolation of the colorless indole-*O*-glycoside indicane which is then hydrolyzed to give indoxyl. The latter undergoes oxidative dimerization to provide indigo. In the 19th century, syntheses of indigo were developed which made the pigment readily available and cheap. Since then, indigo was produced in large scale. The synthesis, derivatization, and application of indigo derivatives have been widely studied [1–3]. Besides their application as dyes, indigo derivatives represent versatile tools for the development of innovative photophysical materials. While unsubstituted (parent) indigo (**1a**) is not a natural product, Tyrian purple (also known as Shellfish purple) is present in nature and represents, besides indigo, an important indigo derivative used as a dye for thousands of years [4].

Several types of cancers, Alzheimer's disease, and Parkinson's disease, cardiovascular diseases, inflammation, AIDS and others have their origin in context with the activities of protein kinases, such as glycogen synthase kinase-3 (GSK-3β) and cyclin-dependent kinases (CDK’s). The phosphorylation of the amino acid moieties of several enzymes is controlled by such protein kinases. Therefore, the investigation of the influence of drugs on protein kinases plays an important role in current medicinal chemistry. Indigo naturalis is a traditional drug, derived from indigo plants, which has been used in China for centuries and also more recently against myelocytic leukemia [5–7]. Indirubin, a red isomer of indigo, is an ingredient of indigo naturalis active against various cancers. Since the 1990s, we are witnessing a renaissance of the chemistry of indirubins because of their activity as potent inhibitors of several kinases, such as GSK-3β and CDK’s [8–10]. In this context, the best CDK2 inhibitory activities were observed for indirubin-derived oximes [11].

Yellow colored isoindigo received a lot of attention as constituent of polymers applied as semi-conducting materials, organic light emitting materials (OLED), and for related applications [12–13]. In addition, there are more and more applications in the field of medicinal chemistry, especially for the treatment of cancer [14–15].

In the course of the renewed interest in the chemistry of indigo, indirubin, and isoindigo in the field of cancer research, *N*-glycosides of these compounds represent promising candidates for drug discovery, because of their improved water solubility, membrane permeability, and improved recognition by the respective receptors [16]. The present article aims to provide an updated review of the chemistry and biological applications of *N*-glycosides of indigo, indirubin, and isoindigo which can be regarded as blue, red, and yellow sugars, respectively.

## Review

### Indigo-*N*-glycosides (blue sugars)

**Scheme 2:**
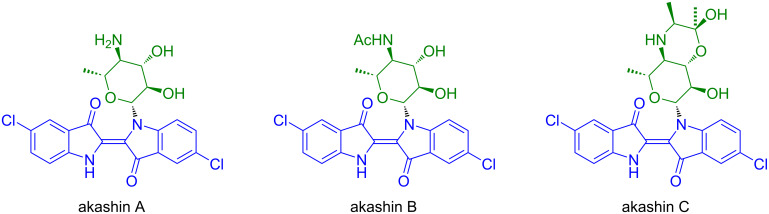
Structures of akashins A–C.

In 2002, Laatsch and Maskey reported the isolation of the akashins A, B and C, indigo-*N*-glycosides, from terrestric *Streptomyces* (Scheme 2) [17–18]. These natural products exhibit an absorption at 618 nm and show a blue color. In contrast to biologically inactive indigo, akashines A–C are active against various human tumor cell lines (CNCL SF268, LCL H460, MACL, colon carcinoma CCL HT29, melanoma MEXF 514L, lung carcinoma LXFA 526L and LXFL 529L, breast cancer MCF-7, kidney tumors PRCL PC3M and RXF 631L) with IC_50_ values of about 2.8 mg mL^−1^ and IC_70_ values of >3 mg mL^−1^.

**Scheme 3:**
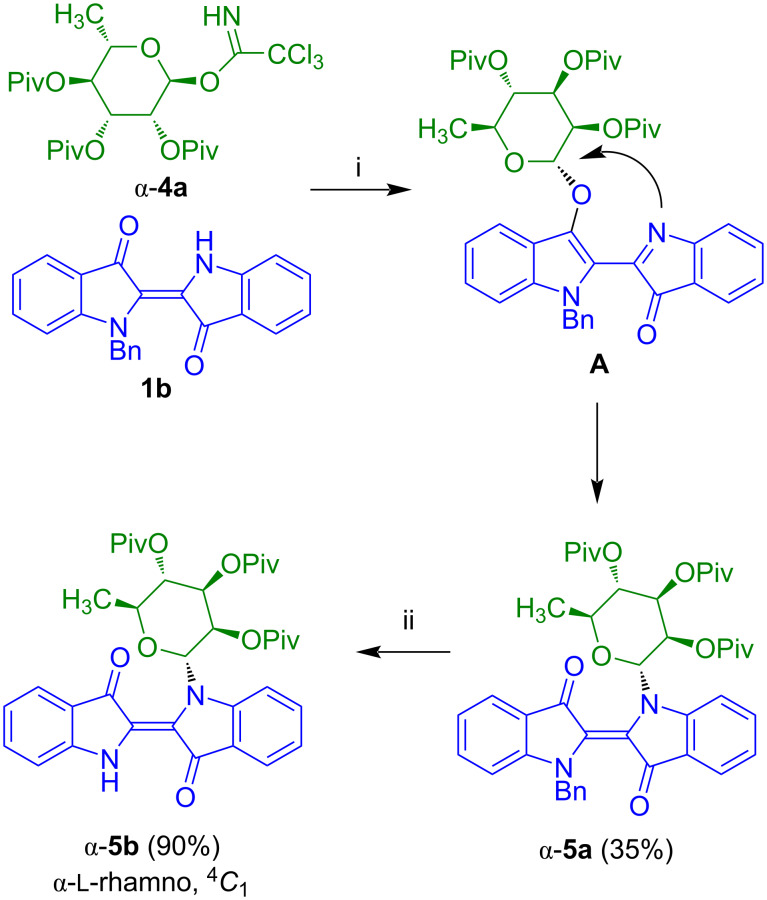
Synthesis of **5b**. Reagents and conditions: i) TMSOTf, 4 Å MS, CH_2_Cl_2_, −20 °C, 1.5 h, then 20 °C, 8–12 h; ii) O_2_, AcOH, 100 °C, 2 h.

In 2005, our group developed a synthesis of indigo-*N*-glycosides (Scheme 3) [19]. The TMSOTf-mediated reaction of readily available *N*-benzylindigo (**1b**) with tri-*O*-pivaloyl-α-ʟ-rhamnosyl trichloroacetimidate (**4a**) initially resulted in the glycosylation of the oxygen atom to give intermediate **A** (−20 °C, 1.5 h). Extension of the reaction time (20 °C, 12 h) afforded *N*-indigoglycoside **5a** which was isolated in 35% yield. The product contained an α-rhamnosyl moiety with ^4^*C*_1_ conformation. The formation of the product can be explained by rearrangement of the rhamnosyl group from the oxygen to the nitrogen atom. Oxidative debenzylation of **5a** afforded **5b** in high yield. Unfortunately, all attempts to remove the pivaloyl protective groups failed. On the other hand, employment of tri-*O*-acetyl-α-ʟ*-*rhamnosyl trichloroacetimidate failed, due to competing formation of orthoester-like amide acetals during the reaction with **1b**. We then focused our work on an alternative synthetic approach (vide infra).

**Scheme 4:**
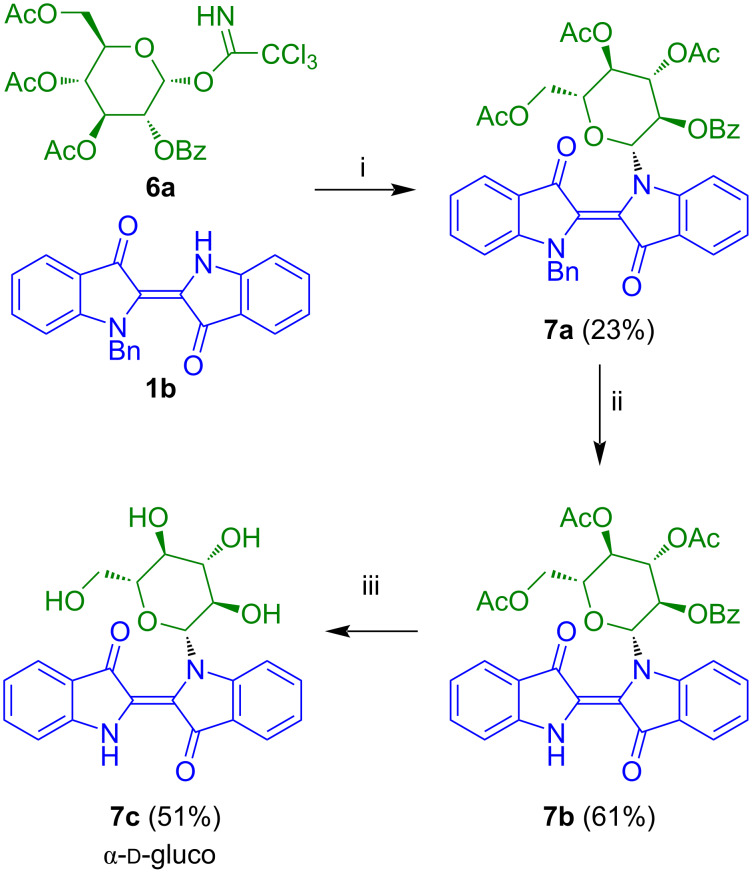
Synthesis of **7c**. Reagents and conditions: i) TMSOTf, 4 Å MS, CH_2_Cl_2_, −18 °C, 3 h; then: TMSOTf, 4 Å MS, CH_2_Cl_2_, 20 °C, 10–12 h; ii) toluene/HOAc 1:1, 50 °C, air, 12–18 h; iii) 1) NaOMe/MeOH, 11 h; 2) Amberlyst 15 (H^+^).

In 2021, Pfretzschner and Unverzagt reported the application of our methodology to the synthesis of indigo-*N*-glucoside **7c** (Scheme 4) [20]. The reaction of **1b** with 3,4,6-tri-*O*-acetyl-2-*O*-benzoyl-α-ᴅ-glucosyl trichloroacetimidate (**6a**) afforded indigo-*N*-glycoside **7a**. The benzoyl group located at OH-2 seemed to be unreactive enough to avoid formation of orthoester-like amide acetals during the *N*-glycosylation with **1b**. Oxidative debenzylation of **7a** gave product **7b** which was transformed to the desired product **7c** by reaction with sodium methoxide and subsequent treatment with acidic ion exchange resin Amberlyst 15.

**Scheme 5:**
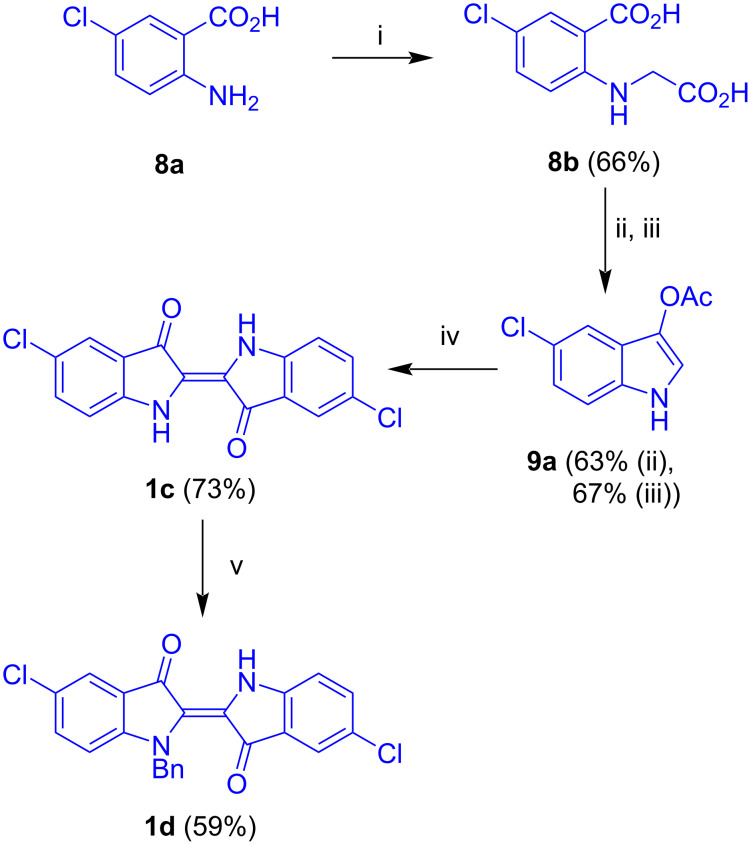
Synthesis of **1d**. Reagents and conditions: i) chloroacetic acid, Na_2_CO_3_, reflux, 6 h; ii) Ac_2_O, NaOAc, reflux, 3 h, 63%; iii) 1) NaOH, reflux, argon, 30 min; 2) Ac_2_O, 0 °C, argon, 45 min, 67% over two steps; iv) NaOH, MeOH, air; v) NaH, DMF, BnBr, 2 h.

The synthesis of akashins A–C was studied next. This required the synthesis of the corresponding indigo and carbohydrate precursors. The base-mediated reaction of 5-chloroanthranilic acid (**8a**) with chloroacetic acid afforded **8b** (Scheme 5) [20]. Acetylation and base-mediated cyclization gave indoxyl **9a** which was transformed to indigo **1c** by oxidative dimerization. Benzylation finally afforded **1d**.

**Scheme 6:**
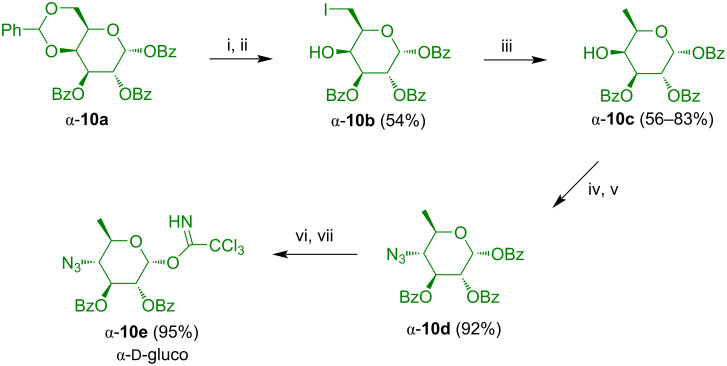
Synthesis of **10e**. Reagents and conditions: i) *p*-TsOH·H_2_O, acetonitrile, MeOH, 1 d; ii) NIS, PPh_3_, DMF, 0–50 °C, 1 d; iii) H_2_, Pd/C (10%), MeOH/HOAc 10:1; iv) Tf_2_O, pyridine, CH_2_Cl_2_, −18 °C, 2 h; v) NaN_3_, DMF, 30 min; vi) hydrazinium acetate, DMF, 20 °C, 3–6 h; vii) Cl_3_CCN, DBU, CH_2_Cl_2_, 0 °C, 2 h.

4,6-Benzylidenation of ᴅ-galactose and subsequent perbenzoylation afforded an anomeric mixture (α:β = 87:13) of tribenzoyl-4,6-benzylidene-ᴅ-galactose from which the pure α-anomer **10a** was separated (Scheme 6) [20]. Cleavage of the acetal and subsequent regioselective replacement of the hydroxy group OH-6 with an iodide by application of the Mukaiyama redox condensation using *N*-iodosuccinic imide (NIS) afforded **10b**. Hydrogenation resulted in defunctionalization to give **10c**. Transformation of OH-4 to a triflate and subsequent reaction with sodium azide afforded gluco-configured product **10d**. The latter was transformed to trichloroacetimidate **10e** by reaction with hydrazine and subsequent treatment with trichloroacetonitrile. The same synthesis was carried out also with the β-configured analogue of **10a**. However, employment of the α-anomer **10a** proved to be advantageous in terms of yield.

**Scheme 7:**
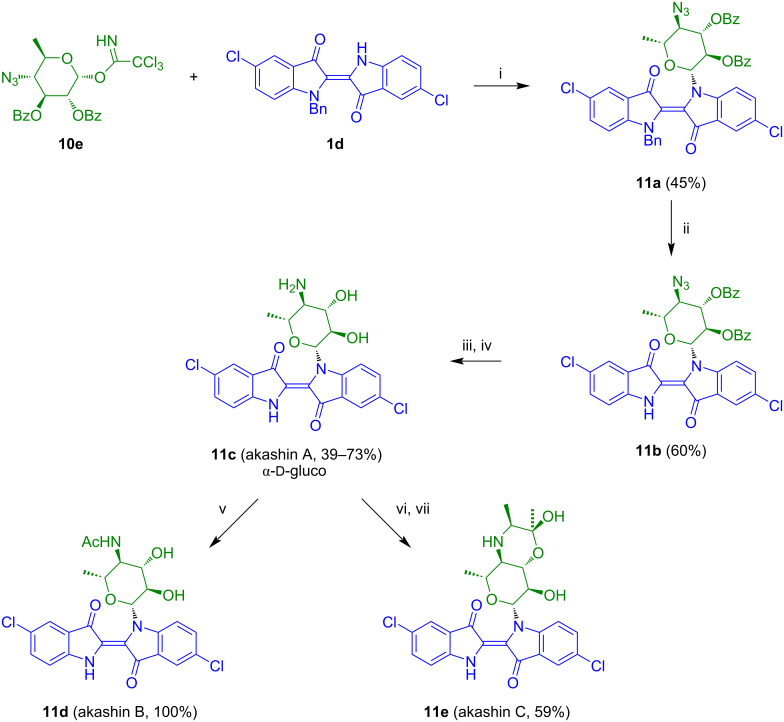
Synthesis of akashins A–C. Reagents and conditions: i) TMSOTf, 4 Å MS, CH_2_Cl_2_, −18 to 20 °C, 15 h; ii) toluene/HOAc 1:1, 60 °C, air, 12–18 h; iii) propanedithiol, DIPEA, MeOH, exclusion of light, 3–4 d, argon; iv) CH_2_Cl_2_/AcOH 99:1, air, 60–90 min; v) Ac_2_O, MeOH, H_2_O, 30–90 min; vi) diacetyl, camphorsulfonic acid (CSA), trimethyl orthoformate, MeOH, 20 °C, 2–3 h; vii) NaCNBH_3_; MeOH, 20 °C, 20–30 min.

The reaction of **1d** with **10e** afforded indigo-*N*-glycoside **11a** (Scheme 7) [20]. Debenzylation gave product **11b** which was transformed to akashin A (**11c**) by reduction of the azide to the amine in the presence of propane-1,3-dithiol and subsequent debenzoylation. Akashin A was transformed to akashins B and C by acetylation and reaction with diacetyl, respectively.

**Scheme 8:**
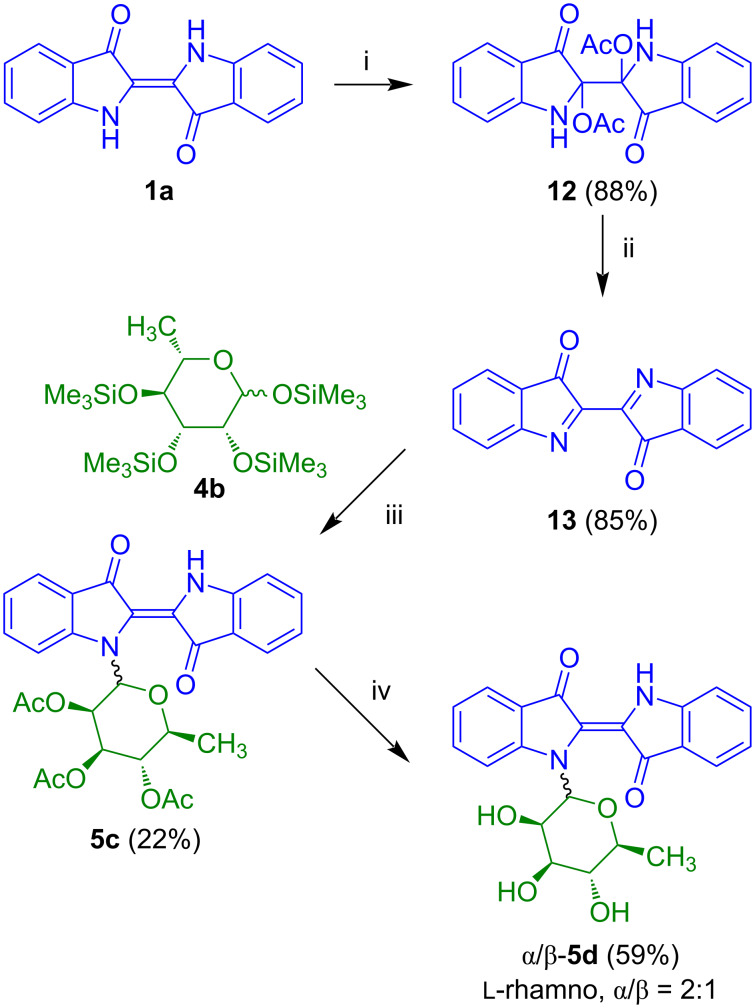
Synthesis of **5d**. Reagents and conditions: i) KMnO_4_, AcOH, high-power-stirring (12.000 rot/min), 20 °C, 3–4 h; ii) pyridine/toluene 1:2, 70 °C, 1 h; iii) 1) CH_2_Cl_2_, 2) Me_3_SiI, 20 °C, 30 min, 3) 0 °C, 30 min; 4) *n*-PrSH, 0→20 °C, 1 h, 5) Ac_2_O/pyridine 3:1, KHF_2_, 70 °C, 3 h; iv) NaO*t-*Bu (15 mol %), MeOH, 20 °C, 4 h.

Because of our difficulties to remove the pivaloyl protective groups of rhamnoside **5b** (Scheme 3), our group developed an alternative synthesis of indigo-*N*-glycosides based on the employment of dehydroindigo (**13**) instead of *N*-benzylindigo (**1b**) (Scheme 8) [21]. Known compound **13** was prepared in two steps from indigo (**1a**). The reaction of **1a** with potassium permanganate afforded product **12** which was transformed to dehydroindigo (**13**) by pyridine-mediated elimination of acetic acid. The reaction of **13** with tetra-*O*-trimethylsilyl-ʟ*-*rhamnopyranose (**4b**) in the presence of trimethylsilyl iodide, addition of *n*-propyl mercaptane with subsequent addition of acetic anhydride, pyridine and KHF_2_ afforded indigo-*N*-rhamnoside **5c** as a 2:1 mixture of α/β-anomers. The relatively low yield is a result of side reactions and decomposition, due to the rather unstable nature of the product. The reaction of **5c** with sodium *tert*-butanolate afforded the desired deprotected indigo-*N*-rhamnoside **5d** (α/β = 2:1). In contrast to the antiproliferative properties of the akashins, **5d** showed no significant activity against human cancer cell lines.

**Scheme 9:**
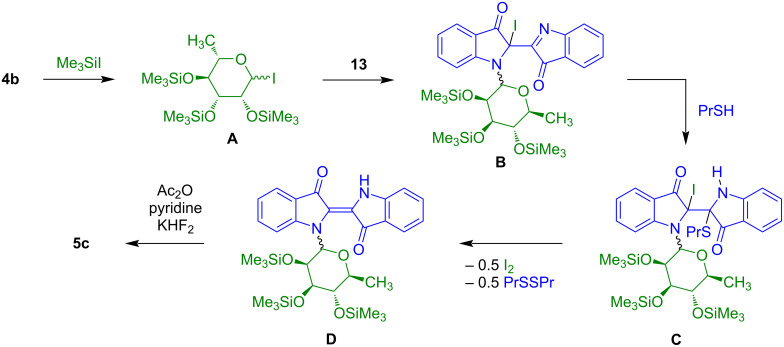
Possible mechanism of the formation of **5c**.

The glycosylation of **13** can be explained as follows (Scheme 9): Reaction of tetra-*O*-trimethylsilyl-ʟ*-*rhamnopyranose (**4b**) with TMSI gave intermediate **A** containing an anomeric iodide. Electrophilic addition of rhamnosyl iodide **A** to one of the two imino groups of **13** gave intermediate **B**. Another electrophilic addition of *n*-propyl mercaptane to the second imino group afforded intermediate **C** which underwent extrusion of iodine and dipropyl disulfide to give intermediate **D**. Subsequent reaction with acetic anhydride, pyridine and KHF_2_ resulted in the replacement of the TMS by acetyl groups which was important for practical reasons (stability during chromatography).

**Scheme 10:**
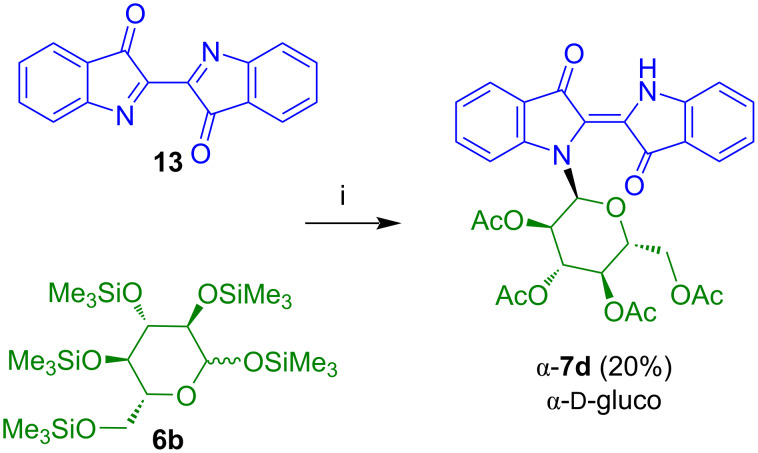
Synthesis of **7d**. Reagents and conditions: i) 1) CH_2_Cl_2,_ 2) Me_3_SiI, 20 °C, 30 min, 3) 0 °C, 30 min, 4) *n-*PrSH, 0→20 °C, 1 h, 5) Ac_2_O/pyridine 3:1, KHF_2_, 70 °C, 3 h.

The reaction of **13** with TMS-protected ᴅ-glucosyl iodide, generated in situ by reaction of penta-*O*-trimethylsilyl-ᴅ-glucose (**6b**) afforded indigo-*N*-α-ᴅ-glucoside **7d**, albeit, in low yield (Scheme 10) [21].

**Scheme 11:**
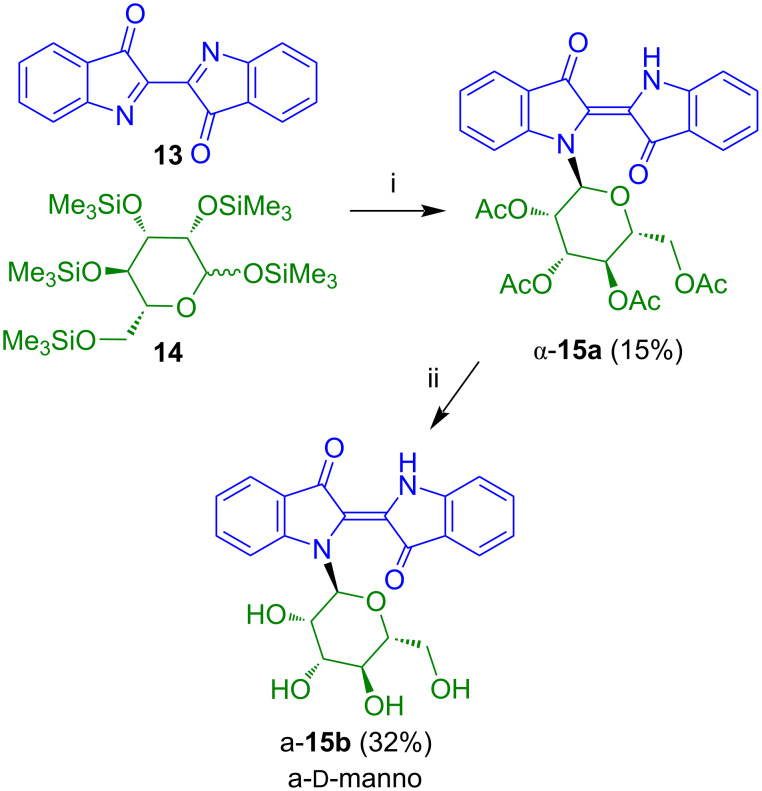
Synthesis of α-**15b**. Reagents and conditions: i) 1) CH_2_Cl_2_, 2) Me_3_SiI, 20 °C, 30 min, 3) 0 °C, 30 min, 4) *n*-PrSH, 0→20 °C, 1 h, 5) Ac_2_O/pyridine 3:1, KHF_2_, 70 °C, 3 h; ii) NaO*t*-Bu (15 mol %), MeOH, 20 °C, 4 h.

The reaction of **13** with TMS-protected ᴅ-mannosyl iodide, generated in situ by reaction of penta-*O*-trimethylsilyl-ᴅ-mannose (**14**) afforded indigo-*N*-mannoside α-**15a**, albeit, in low yield (Scheme 11) [21]. Deacetylation afforded product α-**15b**.

### Indirubin-*N*-glycosides (red sugars)

**Scheme 12:**
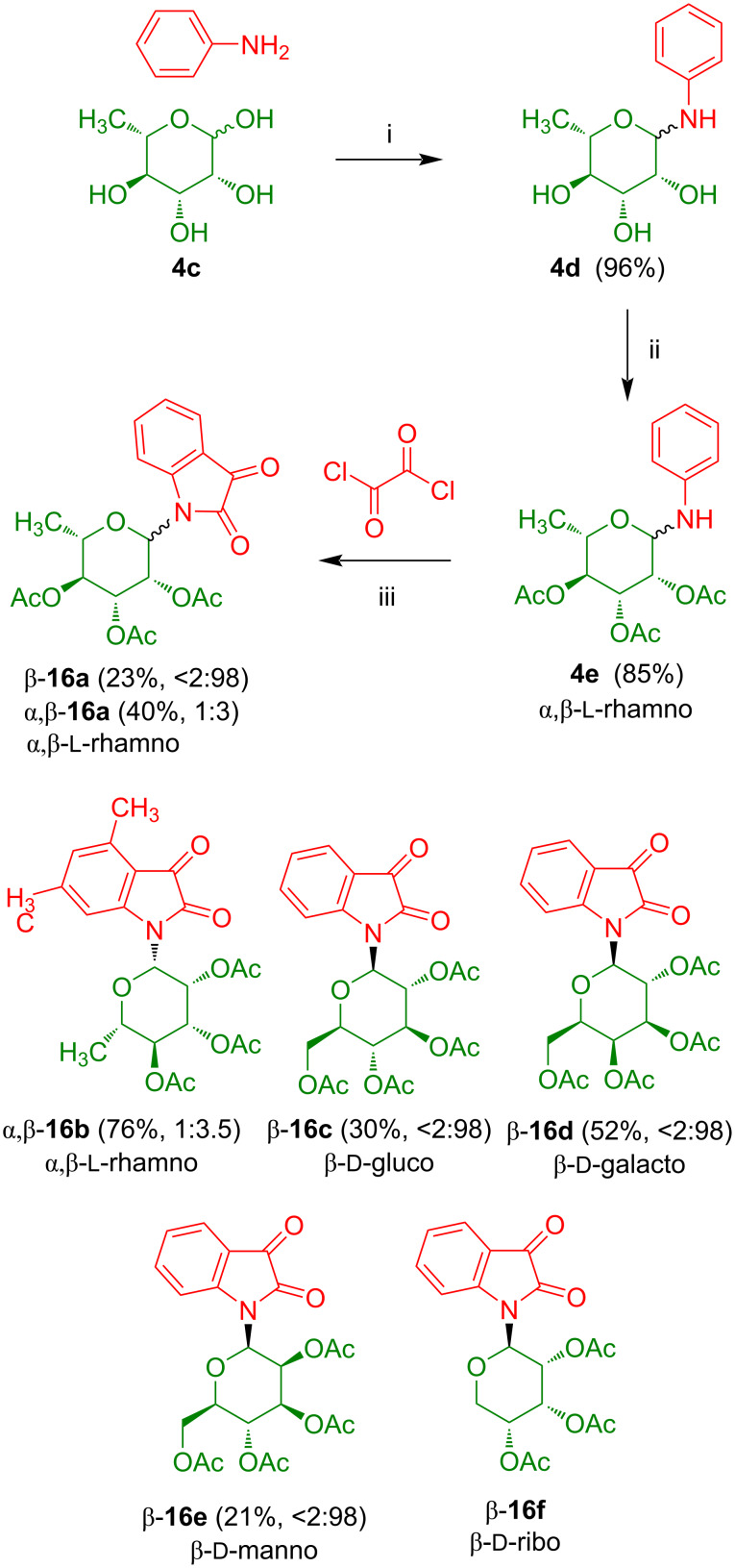
Synthesis of isatin-*N*-glycosides **16a**–**f**. Reagents and conditions: i) PhNH_2_, EtOH, 20 °C, 12 h; ii) Ac_2_O, pyridine, 0 °C, 8–12 h; iii) oxalyl chloride, AlCl_3_, 55 °C, 1.5 h.

In contrast to indigo, indirubin is a non-symmetrical compound containing an amine- and an amide-type nitrogen atom. In our group, we developed a synthesis of indirubin-*N*-glycosides containing the carbohydrate moiety located at the amide-type nitrogen. Isatin-*N*-glycosides **16** were used as key building blocks. The reaction of ʟ-rhamnose (**4c**) with aniline afforded *N*-glycosyl aniline **4d** which was acetylated to give **4e** (Scheme 12) [22]. The AlCl_3_-mediated cyclization of **4e** with oxalyl chloride afforded yellow isatin-*N*-glycoside **16a** as an anomeric mixture from which the pure α- and β-anomer could be separated by chromatography. Likewise, isatin-*N*-glycosides **16b**–**f** were prepared which were all isolated (except from rhamnoside **16b**) as the pure β-anomers.

**Scheme 13:**
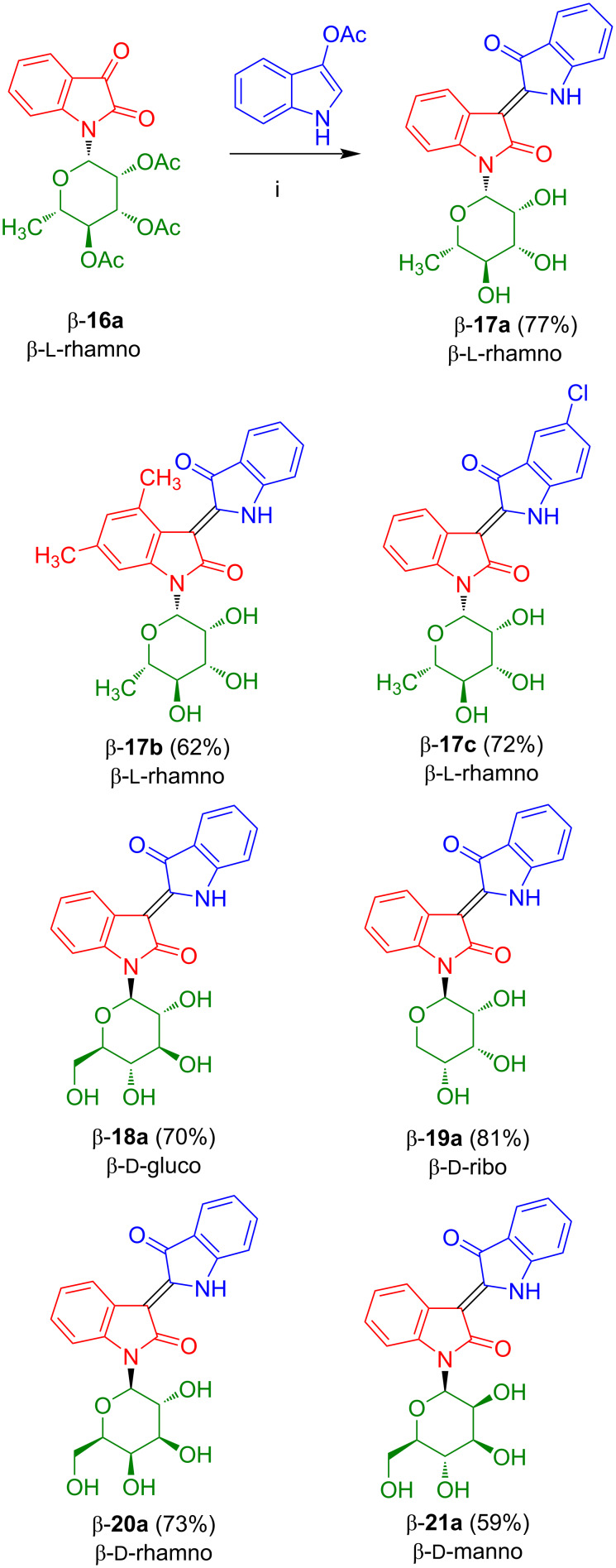
Synthesis of **17**–**21**. Reagents and conditions: i) Na_2_CO_3_, MeOH, 20 °C, 4 h.

The condensation of anomerically pure β-configured β-**16a** with indoxyl acetate, in the presence of an excess of sodium carbonate, afforded β-configured indirubin-*N*-rhamnoside β-**17a** in very good yield as the pure *Z*-configured isomer (Scheme 13) [22]. Gratifyingly, the product, which showed the characteristic red color of indirubin, was directly obtained in its deprotected form. Indirubin-*N*-rhamnoside β-**17a** is significantly more stable than indigo-*N*-rhamnoside **5c**. Likewise, starting with 3,5-dimethylaniline, the β-configured indirubin-*N*-rhamnoside β-**17b** was prepared. Rhamnoside **17c** was prepared by condensation of β-**16a** with 3-acetoxy-5-chloroindole. Anomerically pure indirubin-*N*-glucoside β-**18a**, indirubin-*N*-riboside β-**19a**, indirubin-*N*-galactoside β-**20a**, and indirubin-*N*-mannoside β-**21a** were prepared in good yields starting from the corresponding sugars and anilines [12–13]. In case of the indoxyl moiety, both electron-donating and -withdrawing substituents were tolerated. In contrast, isatin-*N*-glycosides containing electron-withdrawing groups and, thus, the corresponding indirubin-*N*-glycosides could not be successfully prepared at this point (vide infra). In general, anomerically pure isatin-*N*-glycosides had to be employed in order to obtain anomerically pure indirubin-*N*-glycosides. As expected, no epimerization was observed during the base-mediated condensation as no aqueous acid was employed. All products were isolated as the pure *Z*-configured isomers (determination by NMR, the other isomer was not visible, *E*/*Z* < 2:98). The configurations were determined by comparison of chemical shifts of our products with those of non-glycosylated indirubin, by the presence of an intramolecular hydrogen bond N–H···O and by crystal structure analysis. In fact, the chemical shifts of the H-4 proton signals are strongly influenced by the anisotropic effect of the indoxyl carbonyl group resulting in a downfield shift in case of the *Z*-isomers.

**Scheme 14:**
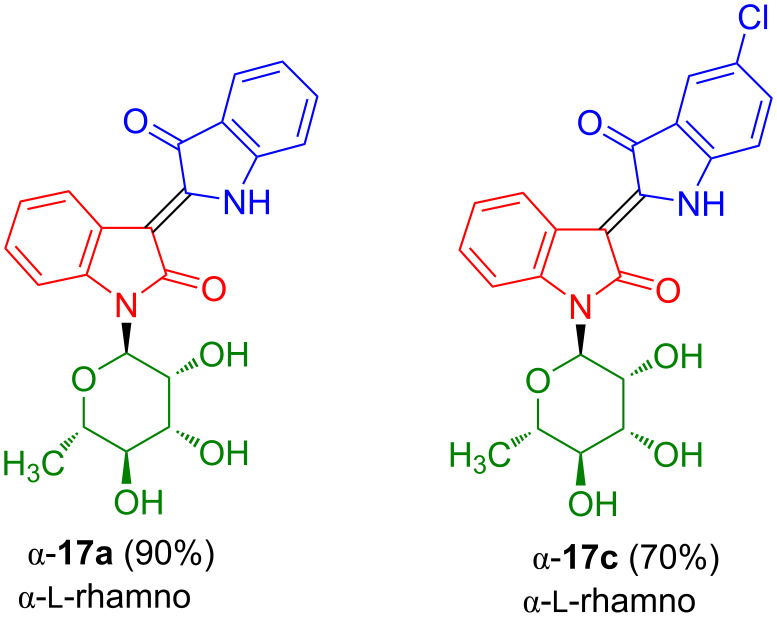
Synthesis of indirubin-*N*-glycosides α-**17a** and α-**17b**.

The reaction of anomerically pure α-configured isatin-*N*-rhamnoside α-**16a** with 3-acetoxyindole and 3-acetoxy-5-chloroindole afforded α-configured indirubin-*N*-rhamnosides α-**17a** and α-**17c**, respectively (Scheme 14) [23].

**Scheme 15:**
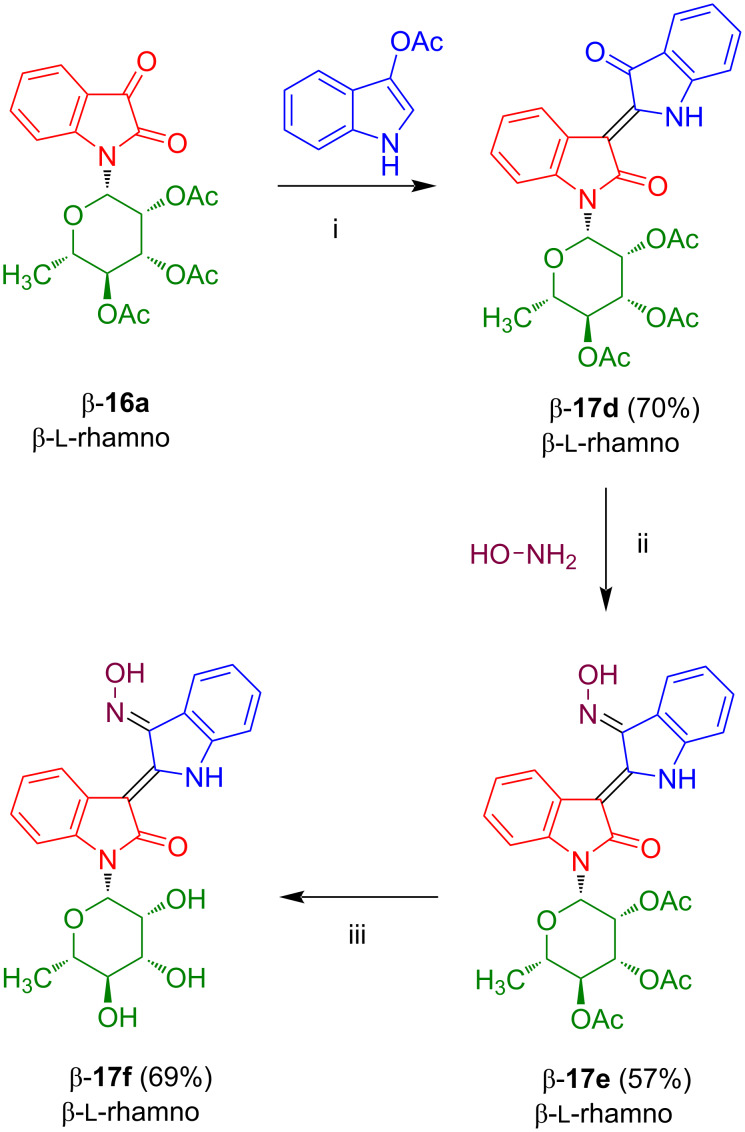
Synthesis of β-**17f**. Reagents and conditions: i) 1) Na_2_CO_3_, MeOH, 20 °C, 4 h, 2) Ac_2_O/pyridine 1:1, 0 °C, 12 h; ii) H_2_NOH·HCl, pyridine, 90 °C, 7 h; iii) KO*t-*Bu, MeOH, 20 °C, 12 h.

Water solubility plays an important role with regard to the pharmacological activity. It was previously shown that the water solubility and anticancer activity of non-glycosylated indirubins can be improved by conversion of the keto to an oxime group [8–11]. Therefore, we studied the synthesis of oximes of indirubin-*N*-glycosides. The reaction of isatin-*N*-rhamnoside β-**16a** with 3-acetoxyindole and subsequent acetylation afforded indirubin-*N*-rhamnoside β-**17e** which was subsequently deprotected to give oxime β-**17f** (Scheme 15) [23].

In collaboration with Michael Lalk and Patrick Bednarski (University of Greifswald), the antiproliferative activities of indirubin-*N*-rhamnosides **17a**–**c**,**f**, indirubin-*N*-glucoside β-**18a**, indirubin-*N*-galactoside β-**20a**, and indirubin-*N*-mannoside β-**21a** were studied. In this context, four human cancer cell lines were employed, namely, bladder (5637), small cell lung (A-427), esophageal (Kyse-70), and breast (MCF-7) [23]. Rhamnosides α-**17a** and β-**17b** showed excellent activities against the human breast cancer cell line MCF-7. In general, the best activities were observed for indirubins containing no additional substituent. The carbohydrate moiety also has an influence on the antiproliferative activity. The best activities were observed for rhamnosides, while the lowest activity was observed for galactoside β**-20a**. The configuration of the anomeric carbon atom of the rhamnoside did not show a major influence as very good activities were observed for both α-**17a** and β-**17a**. It is important to note that the anticancer activity of all indirubin-*N*-glycosides, in particular of rhamnosides α-**17a** and β-**17a**, was considerably higher as compared to the activities of previously reported non-glycosylated indirubins [23–24]. A low activity was observed for oxime β-**17f** which is surprising, as good activities were previously reported for indirubin oximes as compared to other non-glycosylated indirubins.

**Scheme 16:**
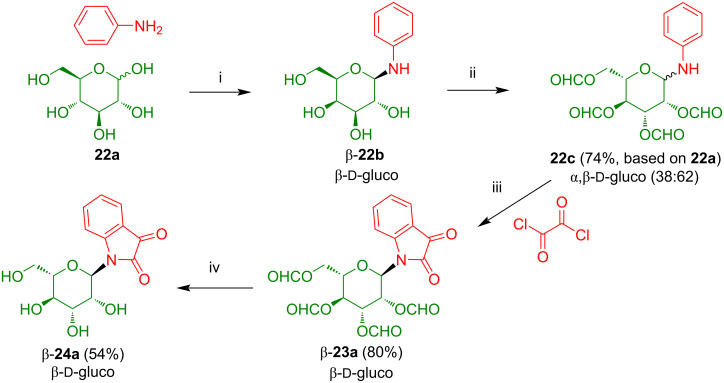
Synthesis of β-**24a**. Reagents and conditions: i) *n-*PrOH, H_2_O, formic acid (buffer, 100 mM), 2 h, 65 °C; ii) pyridine, DMF, formic acid, Ac_2_O, ethyl formiate, 4 h, −40 °C; iii) 1) CH_2_Cl_2_, oxalyl chloride, 4 h, −20 °C, 2) ClC_6_H_5_, TiCl_4_, 5 h, 85 °C; iv) THF, MeOH, NH_3_, 30 min, 20 °C.

As mentioned above, the synthesis of acceptor-substituted isatin-*N*-glycosides by Lewis acid-mediated cyclization of the corresponding acetyl-protected *N*-glycosyl anilines with oxalyl chloride was not possible. The failure of this reaction can be explained by electronic deactivation of the aromatic ring and steric hindrance which resulted in competing Claisen-type side-reactions of the acetyl groups. However, reactions of pivaloyl-protected *N*-glycosyl anilines also proved to be unsuccessful, which pointed to steric reasons. Therefore, we studied in our group the use of the less sterically hindered formyl instead of acetyl or pivaloyl protective groups. The reaction of glucose (**22a**) with aniline afforded anomerically pure *N*-glycosyl aniline β-**22b** which was formylated to give **22c** in 74% yield (Scheme 16) [24]. Product **22c** resides as a mixture of α- and β-anomers, because an anomeric equilibrium was activated during the acid-mediated formylation. The cyclization of **22c** with oxalyl chloride, carried out under forcing conditions (TiCl_4_, chlorobenzene, 85 °C), afforded anomerically pure β-configured isatin-*N*-glucoside β-**23a** in 80% yield. The product was formed as the pure β-anomer, because the anomeric equilibrium was again active in the presence of the Lewis acid and the β-anomer is thermodynamically favored because of the steric demand of the isatin moiety and the α-anomeric form would result in a disfavorable 1,3-diaxial interaction between the isatin and the formyl group located at carbons C-1 and C-3, respectively. Deprotection with ammonia afforded the deprotected glucoside β-**24a** in anomerically pure form.

**Scheme 17:**
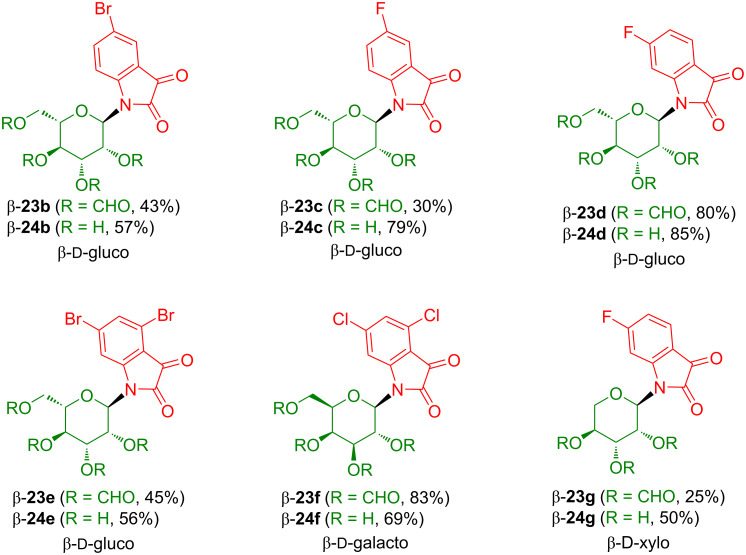
Synthesis of isatin-*N*-glycosides **23b**–**g** and **24b**–**g**.

Following the methodology described above, acceptor-substituted isatin-*N*-glucosides **23b**–**f** and xyloside **23g** were prepared in 25–83% yields (Scheme 17) [24]. All products were formed as the β-anomers. The yield of product **23e** derived from *m*-fluoroaniline was significantly higher as compared to product **23c** derived from *p*-fluoroaniline which can be explained by the π-donating effect of fluorine which facilitates the cyclization via the *ortho*-position in case of *m*-, but not of *p*-fluoroaniline. It is worth to be noted that the formation of **23d** proceeded with excellent regioselectivity via carbon C-6 rather than C-2 of the 3-fluoroaniline moiety, presumably due to steric reasons. Deprotection of the isatin-*N*-glucosides **23b**–**f** and xyloside **23g** proceeded uneventfully and afforded products **24b**–**g** in 50–85% yields. All products were isolated as the β-anomers.

**Scheme 18:**
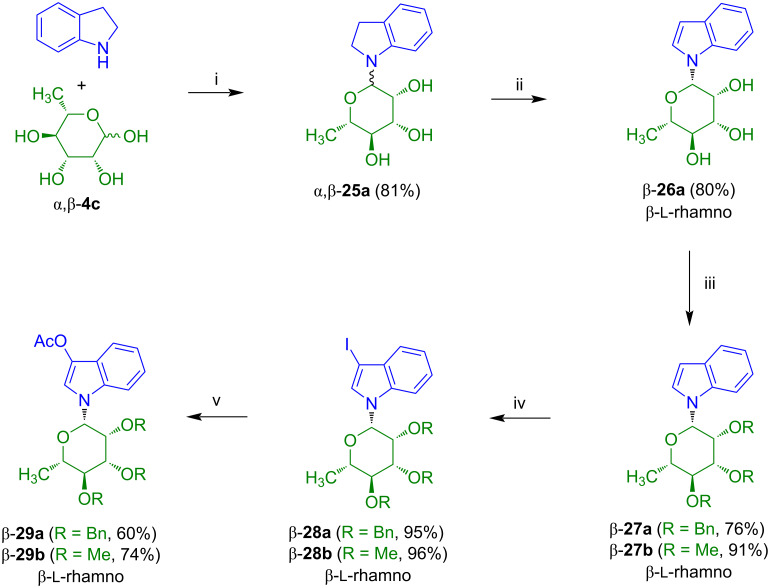
Synthesis of β-**29a**,**b**. Reagents and conditions: i) EtOH, 20 °C, 12 h; ii) DDQ, dioxane, 20 °C, 12 h; iii) BnBr (for β-**27a**) or MeI (for β-**27b**), NaH, DMF, 0→4 °C, 12 h; iv) I_2_, NaOH, DMF, 20 °C, 1 h; v) AgOAc, AcOH, 80 °C, 4 h.

In our group, we also studied the synthesis of indirubin-*N*-glycosides containing the carbohydrate moiety located at the amine-type nitrogen atom (Scheme 18) [25]. For this purpose, we decided to react isatins with *N*-glycosylated indoxyls which were entirely unknown at that time. The reaction of ʟ-rhamnose α/β-**4c** with indoline afforded α/β-**25a** (β:α ≈ 4:1). Dehydrogenation of the latter with DDQ afforded the anomerically pure indol-*N*-glycoside β-**26a** which upon benzylation and methylation gave products β-**27a** and β-**27b**, respectively. Iodination gave products β-**28a** and β-**28b**, however, due to the basic reaction conditions (I_2_, NaOH, DMF), ether rather than ester protective groups had to be employed. The reaction of the 3-iodoindoles β-**28a** and β-**28b** with an excess of silver acetate under mild acidic conditions afforded 3-acetoxyindol-*N*-rhamnosides β-**29a** and β-**29b**, respectively.

**Scheme 19:**
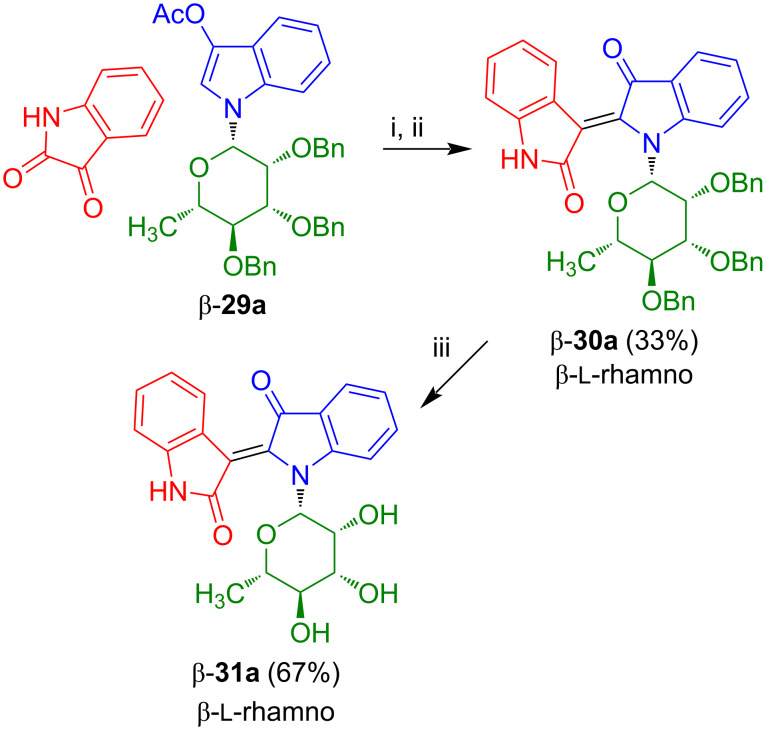
Synthesis of β-**31a**. Reagents and conditions: i) Na_2_SO_3_, dioxane, H_2_O, 110 °C, 2 d; ii) piperidine, benzene, 80 °C, 2 h; iii) BBr_3_, CH_2_Cl_2_, −78 °C, 3.5 h.

The application of the conditions used for the synthesis of indirubin-*N*-glycoside **17a** (Scheme 13) failed for the reaction of β-**29a** and β-**29b** with isatin. Likewise, various other conditions for base- or acid-mediated deacetylation and aldol-type condensation, typically used for the synthesis of non-glycosylated indirubins, proved to be unsuccessful. However, the acetyl group of β-**29a** could be selectively removed under slightly basic and reductive conditions (Na_2_SO_3_, dioxane, H_2_O) to give the desired free indoxyl-*N*-glycoside. Due to its instability in the presence of air, the crude material was directly used for the reaction with isatin to give the desired indirubin-*N*-rhamnoside β-**30a**, albeit, in low yield (Scheme 19) [25]. Cleavage of the benzyl groups using boron tribromide afforded β-**31a**. In contrast to indirubin-*N*-rhamnoside **17a**, its isomer β-**31a** proved to be rather unstable. This fact was already earlier observed for indigo-*N*-rhamnoside **5d** and again suggests that compounds containing the sugar moiety attached to the amine-type nitrogen are less stable than the corresponding compounds containing the sugar moiety attached to the amide-type nitrogen atom. Unfortunately, the synthesis of β-**31a** was not a general method for the synthesis of indirubin-*N*-glycosides.

### Analogues of indirubin-*N*-glycosides

#### Thioindirubin-*N*-glycososides

**Scheme 20:**
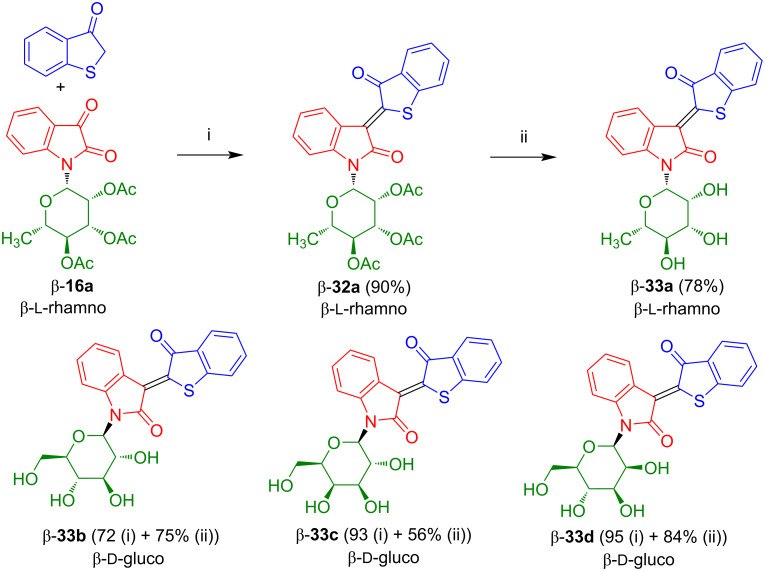
Synthesis of **33a**–**d**. Reagents and conditions: i) Ac_2_O, AcOH, NaOAc, 80 °C, 1 h; ii) 1) NaOMe, anhydrous MeOH, 20 °C, 2) 1% HCl in anhydrous MeOH, 20 °C. The yields of **33b**–**d** refer to the yields of the condensation and the deprotection step for each compound.

In our group, we studied the reaction of isatin-*N*-glycoside **16a** with thiaindan-3-one which afforded red colored thioindirubin-*N*-rhamnoside β-**32a** in very good yield and with excellent *Z*-selectivity (Scheme 20) [26]. Deprotection of β-**32a** afforded β-**33a** in very good yield. Likewise, glucoside β-**33b**, galactoside β-**33c**, and mannoside β-**33d** were prepared. All products were isolated as the pure *Z*-configured isomers (determination by NMR, the other isomer was not visible, *E*/*Z* < 2:98). The configurations were determined by comparison of chemical shifts of our products with those of non-glycosylated thioindirubin and by crystal structure analysis. As mentioned above for the indirubins, the chemical shifts of the H-4 proton signals are strongly influenced by the anisotropic effect of the carbonyl group resulting in a downfield shift in case of the *Z*-isomers.

In collaboration with Manfred Kunz (University of Leipzig), it was shown that all four compounds exhibit excellent antiproliferative activity against the malignant melanoma cell lines MeI-19, SK-MeI-29, SK-MeI-103, and SK-MeI-147. The compounds also induce apoptosis of the cancer cell lines which was investigated in collaboration with Jürgen Eberle (Charité Berlin) [27]. In fact, melanoma is a dangerous type of skin cancer which can be life threatening. Excellent IC_50_ values were observed for β-**33d** and other thioindirubin-*N*-glycosides. In contrast, indirubin-*N*-rhamnoside β-**17a**, lacking the sulfur atom, proved to be inactive against melanoma cell lines. In the future, it will be interesting to study the synthesis of acceptor-substituted thioindirubin-*N*-glycosides by reaction of thiaindan-3-one with acceptor-substituted isatin-*N*-glycosides **23b**–**g**. Indirubin- and thioindirubin-*N*-rhamnosides played an important role in an interdisciplinary project to understand the generation, development, and action of melanoma with the goal to develop new ways of clinical treatment of this dangerous disease. In this context, special emphasis was given to cell lines A-375 and SK-Mel-28 [27].

**Scheme 21:**
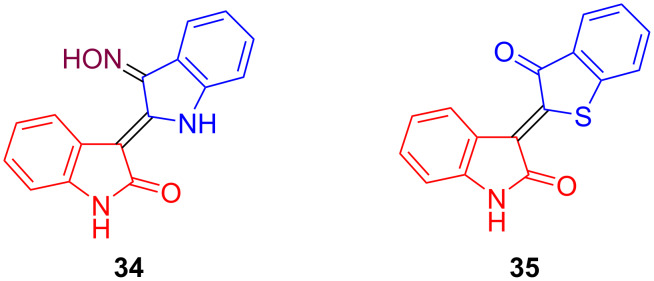
Indirubins **34** and **35**.

As mentioned above, non-glycosylated indirubins act as a ligand for the aryl hydrocarbon receptor (AhR) and also directly inhibit several cyclin-dependent kinases (CDK). A problem is generally the low water solubility of these compounds. The commercially available indirubin-3’-monoxime (**34**) shows a better solubility, due to the presence of the oxime group (Scheme 21). In fact, it represents one of the most active non-glycosylated indirubin derivatives against cancer [11,28]. It has been reported that the free NH function of the indirubin is crucial for CDK inhibition. The reaction of isatin with thiaindan-3-one has been reported to give thioindirubin **35** in very good yield as the pure *Z*-configured isomer [29]. For reasons of comparison, non-glycosylated indirubins **34** and **35** were prepared in our group.

Cutaneous squamous cell carcinoma (CSCC) is the second most common non-melanoma skin cancer (NMSC) after basal cell carcinoma (BCC). CSCC is responsible for 20% of cutaneous diseases and about 75% of all deaths are due to skin cancer, excluding melanoma. Due to the high incidence of CSCC and of actinic keratosis, there is a need of new therapeutic interventions. Thioindirubin-*N*-glycosides β-**33b** and β-**33d** show an excellent cytotoxic activity against melanoma and squamous cell carcinoma cells, lung cancer and glioblastoma cells [30]. It was shown in a WST-1 assay that these compounds exhibit a concentration-dependent inhibition of the metabolic activity of A375 and A431 cells, while the non-glycosylated derivative **34** proved to be inactive. The activities of β-**33b** and **34** on the cell viability was independently studied by using crystal violet as a dye for viable cells following a 48 h incubation of A375 melanoma cells and A431 squamous carcinoma cells. Interestingly, a concentration-dependent cytotoxicity was again observed for β-**33b**, but not for **35** in both cell lines. The IC_50_ values of β-**33b** were similar to those obtained by the WST-1 assay. A related proapoptotic effect was not observed for **35** in A375 cells. However, in case of A431 cells, a significant increase in caspase activity was observed for **35**, but at a much lower level as compared to β-**33b**.

For compound β-**33b**, the mitochondrial properties and cell viability of melanoma (A375) and squamous cell carcinoma cells (A431) of the skin were studied [31]. Glucoside β-**33b** resulted in a decrease of the cell viability. In addition, activation of caspases-3 and -7 and inhibition of colony formation were observed. Likewise, a concentration-dependent decrease of both the basal and ATP-linked oxygen consumption rate and of the capacity of oxidative respiration were observed at the mitochondrial level in the presence of compound β-**33b**. In addition, the morphology of the mitochondria was changed. The presence of β-**33b** provoked a significant upregulation of the enzyme heme oxygenase-1. In conclusion, mitochondria are attacked by compound β-**33b** in the context of its cytotoxic activity against skin cancer cells. Aglycon **35** again proved to be inactive in all assays.

It was mentioned above, that the free NH function of indirubin is important for CDK inhibition. We have shown in our group by enzyme studies that glycoside β-**33b**, lacking the free NH-function, does not act as a CDK inhibitor, despite its high activity against melanoma cells and other cancers [32]. These results point to a completely different mode of action. In fact, the exact function of glycosylation is not clear. However, it might be assumed that the carbohydrate moiety increases the water solubility of the indirubin which results in a higher bioavailability of the drug. It might be speculated that the carbohydrate moiety is later hydrolytically cleaved in the cell to release the indirubin which then acts as a CDK inhibitor.

A synergistic effect of plasma-activated medium (PAM) and β-**33b** on human skin cancer cells was observed [32–33]. With regard to viability, adhesion capacity, apoptosis and G2/M cell cycle arrest, especially in A375 cells, glycoside β-**33b** alone showed a stronger anticancer effect as compared to oxime **34**. PAM significantly increased these effects in skin cancer cells. In contrast, no effect was observed for non-glycosylated thioindirubin **35** alone or in combination with PAM. It is anticipated that PAM activates channels of the membrane of melanoma cells to allow the antiproliferative compound (β-**33b**) to enter the cell. Thus, β-**33b** combined with PAM might be developed to a new and promising method of therapeutic intervention. This concept of combination of PAM with drugs could also be applied to other small compounds developed in our laboratory [34].

#### Selenoindirubin-*N*-glycososides

**Scheme 22:**
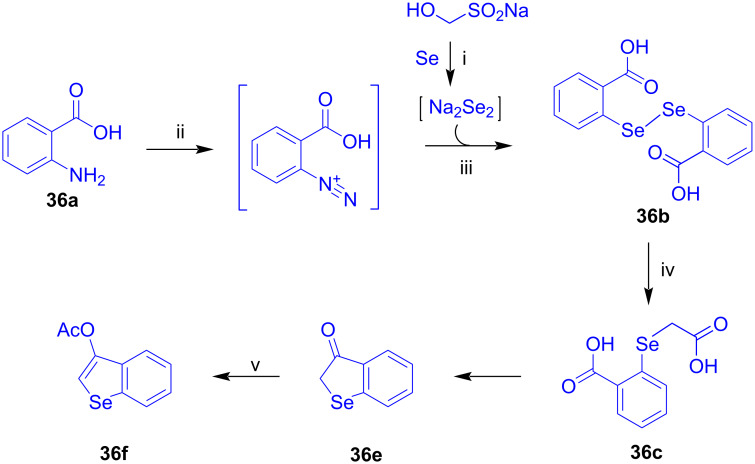
Synthesis of **36f**. Reagents and conditions: i) NaOH, H_2_O, 20 °C, 5 h; ii) HCl, NaNO_2_, H_2_O, −14 °C; iii) 1) Na_2_Se_2_, K_2_CO_3_, H_2_O, 0→100 °C, 2) HCl; iv) 1) Zn, NaOH, H_2_O, 100 °C, 0.5 h, 2) BrCH_2_COONa, 100 °C, 0.5 h, 3) HCl; v) Ac_2_O, pyridine, 90 °C, 6 h.

3-Acetoxybenzo[*b*]selenophene (**36f**) was prepared in our group according to a literature procedure (Scheme 22) [35–37]. The synthesis is based on the reaction of disodium diselenide with the diazonium salt derived from anthranilic acid (**36a**) to give diselenide **36b**, followed by reaction with the sodium salt of bromoactic acid, cyclization, and acetylation.

**Scheme 23:**
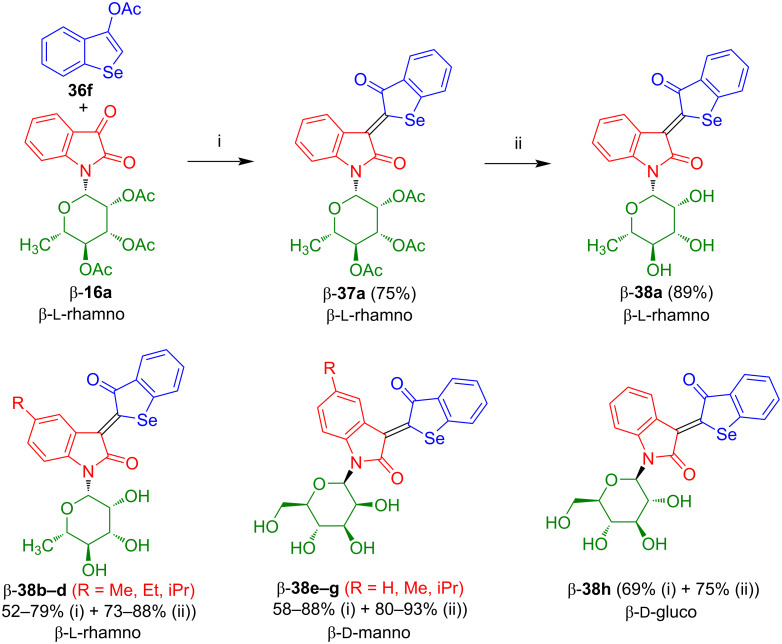
Synthesis of **38a**–**h**. Reagents and conditions: i) 1) 0.1 equiv NaOMe, MeOH, 20 °C, 15–20 min, 2) HOAc, NaOAc, Ac_2_O, 80 °C, 3–4 h; ii) NaOMe (cat.), MeOH/THF 2:1, 20 °C, 7 h. The yields of **38b**–**h** refer to the yields of the condensation and the deprotection step for each compound.

In our group, we investigated the reaction of isatin-*N*-rhamnoside **16a** with **36f** which afforded selenoindirubin-*N*-rhamnoside β-**37a** in good yield and with excellent *Z*-selectivity (Scheme 23) [38]. Deprotection of β-**37a** gave β-**38a** in very good yield. Likewise, rhamnosides β-**38b**–**d**, mannosides β-**33e**–**g**, and glucoside β-**38h** were prepared.

**Scheme 24:**
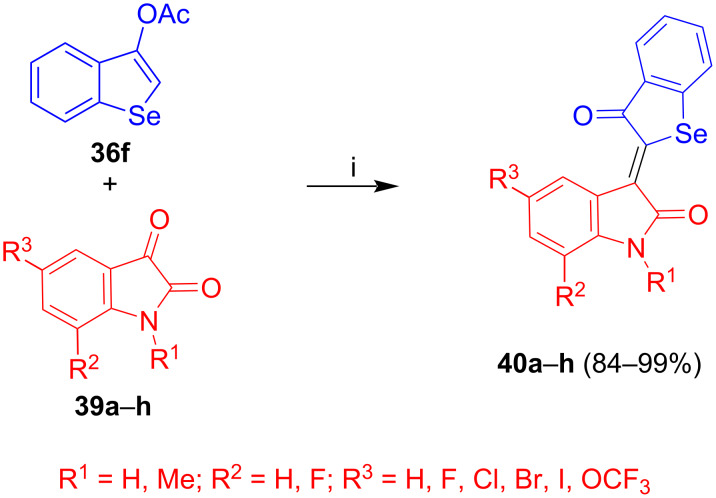
Synthesis of **40a**–**h**. Reagents and conditions: i) method A: EtOH/THF, cat. KO*t*-Bu, 20 °C, 3–4.5 h; method B: 1) MeOH, NaOMe (0.1 equiv), 20 °C, 15–20 min, 2) HOAc (0.1 equiv); 3) cat. piperidine, EtOH, 1.5 h.

The reaction of **36f** with isatins **39a**–**h** afforded novel non-glycosylated selenoindirubins **40a**–**h** (Scheme 24) [38].

In collaboration with Jürgen Eberle and his team it was shown that selenoindirubin-*N*-glycosides β-**38a**–**h** showed antiproliferative activity against lung cancer cell lines H157 [38]. The antiproliferative activity against melanoma cells was accompanied by induced apoptosis in combination with the death ligand TRAIL (TNF-related apoptosis-inducing ligand). The death ligand TRAIL is a promising strategy for cancer treatment. In contrast to non-glycosylated thioindirubin **35**, a significant activity was observed also for non-glycosylated selenoindirubin **40h**. This might be due to the fact that selenoindirubin-*N*-glycosides show a considerably better solubility during the biological assays. Although the activities were, in general, only moderate, the results were promising in the sense that the activity was (again) concentration-dependent. It is important to note, however, that the results of the antiproliferative activities of selenoindirubin-*N*-glycosides β-**38a**–**h** were not directly comparable with those of thioindirubin-*N*-glycosides β-**33a**–**d**, because different cell lines were employed.

#### Oxoindirubin-*N*-glycososides

**Scheme 25:**
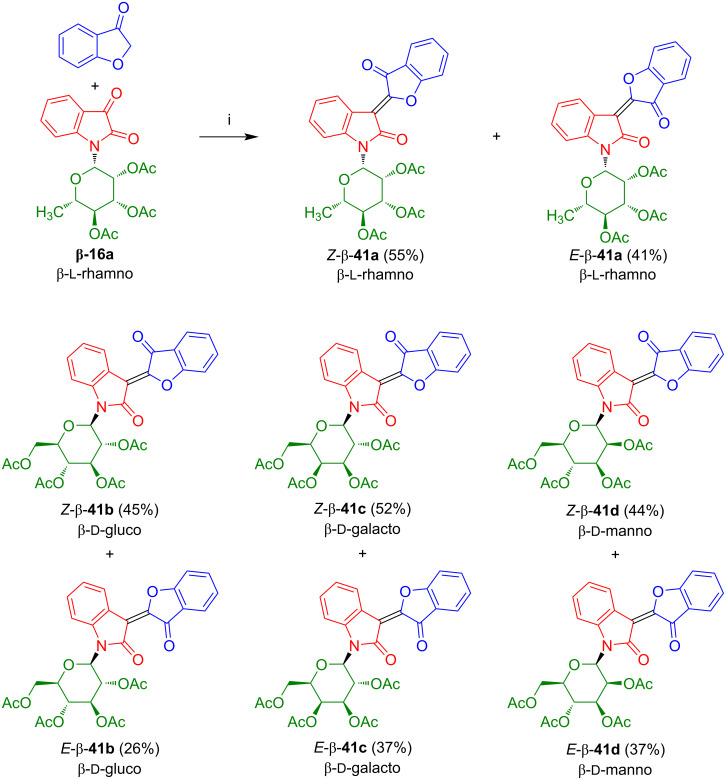
Synthesis of **41a**–**d**. Reagents and conditions: i) Ac_2_O, AcOH, NaOAc, 80 °C, 1 h.

We studied in our group the reaction of isatin-*N*-rhamnoside **16a** with cumaran-3-one which afforded the desired oxoindirubin-*N*-rhamnoside as a separable mixture of *Z*-β-**41a** (55%) and *E*-β-**41a** (41%) (Scheme 25) [39]. Likewise, glucoside β-**41b**, galactoside β-**41c**, and mannoside β-**41d** were prepared and the two geometric isomers of each product were separated and isolated in pure form. However, the deprotection failed under various conditions, due to decomposition by retro-aldol reaction. Interestingly, while indirubin-*N*-glycosides **17**, thio- and selenoindirubin-*N*-glycosides **33** and **38** were formed as pure *Z*-isomers, oxoindirubin-*N*-glycosides **41** were formed as mixtures of *E-* and *Z*-isomers. The isolated yields of *Z*-β-**41a** (55%) and *E*-β-**41a** (41%) are in accordance with the relative thermodynamic stability of the two geometric isomers. According to DFT calculations, the Z-isomer is slightly energetically favored by 14.88 kJ/mol. The configurations were determined by comparison of chemical shifts of our products with those of non-glycosylated indirubin, by the presence of an intramolecular hydrogen bond N–H···O and by crystal structure analysis. As mentioned above for the indirubins and thioindirubins, the chemical shifts of the H-4 proton signals are strongly influenced by the anisotropic effect of the coumaranone carbonyl group resulting in a downfield shift in case of the *Z*-isomers. The different *E*/*Z*-selectivity in case of oxoindirubins as compared to other indirubin derivatives can be explained, on the one hand, by the absence of the favorable intramolecular hydrogen bond which is present in case of indirubins **17**. However, this does not explain the difference to thio- and selenoindirubins **33** and **38**. In general, the isomer containing the two carbonyl groups on opposite sides of the central double bond is thermodynamically favored, due to electrostatic repulsion. However, the presence of the ring oxygen in case of the oxoindirubins **41** seems to decrease this unfavorable dipol situation in case of the *E*-configured isomers. It is likely, that an *E*/*Z* isomerization takes place under acidic conditions. The barrier of this equilibrium is most likely lowered by the presence of the ring oxygen as it represents a π-donor. Although nitrogen, as present in indirubins **17**, is an even better donor than oxygen, the stability of the intramolecular hydrogen bond overrules this effect. Sulfur and selenium are very weak π-donors. It was not studied so far whether the *E*/*Z* ratio of indirubin derivatives can be influenced by UV irradiation.

**Scheme 26:**
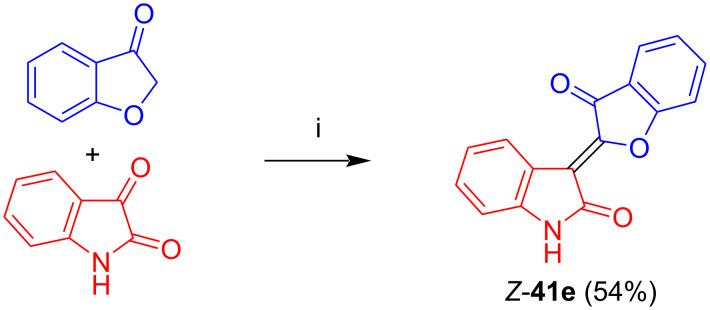
Synthesis of **41e**. Reagents and conditions: i) AcOH, NaOAc, 110 °C, 24 h.

Kornienko et al. reported the synthesis and antiproliferative activity of non-glycosylated oxoindirubin *Z*-**41e** in 54% yield by reaction of cumaran-3-one with isatin under conditions similar to those employed by us (Scheme 26) [40]. However, the reaction time was much longer (24 instead of 1 h). Interestingly, in contrast to *N*-glycosides **41a**–**d**, only one geometrical isomer was formed. This result is surprising, as the carbohydrate moiety is located far away from the double bond and unlikely exerts a stereochemical effect. As the aldol condensation is irreversible once the double bond is formed, a thermodynamic control of the *E/Z*-ratio and influence of the reaction time is unlikely. However, the reported formation of **41e** as a single isomer has to be treated with some care. In the publication, no supporting information and compound characterization was given. In addition, the yield of **41e** (54%) is in a range which does not really exclude the formation of the *E*-configured isomer as a side-product. In fact, we observed for the formation of *Z*-configured *N*-glycosides **41a**–**d** yields in the range of 44–55%, while the corresponding *E*-configured isomers were isolated in 26–41% yields. In any case, compound *Z*-**41e** was reported to exhibit a strong in vitro antiproliferative activity against breast cancer cells MCF-7 and melanoma mouse cells B16F10. In contrast, the activity against melanoma cells SKMEL-28 was only moderate.

#### Carboindirubin-*N*-glycososides

**Scheme 27:**
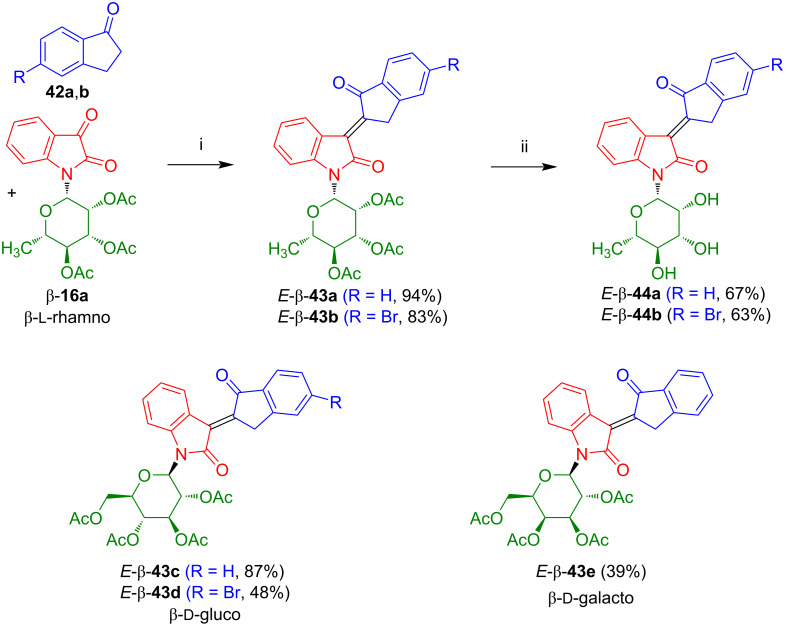
Synthesis of *E*-β-**43a**–**e** and *E*-β-**44a**,**b**. Reagents and conditions: i) 1) NEt_3_, EtOH, 20 °C, 12 h, 2) DMAP, NEt_3_, MsCl, CH_2_Cl_2_, 0 to 20 °C, 20 h; ii) HCl in MeOH (1%), 20 °C, 12 h.

The reaction of isatin-*N*-rhamnoside **16a** with indan-1-ones **42a**,**b**, carried out in our group, afforded *E*-configured carboindirubin-*N*-rhamnosides *E*-β-**43a**,**b** in very good yields (Scheme 27) [39,41]. In this reaction, a solution (EtOH) of the starting materials and NEt_3_ was stirred at 20 °C to give the corresponding aldol products. The latter were treated, without chromatographic purification, with MsCl (methanesulfonyl chloride), NEt_3_, and DMAP to give the desired products *E*-β-**43a**,**b**. Similarly, glucosides *E*-β-**43c**,**d** and galactoside *E*-β-**43e** were obtained. In contrast to oxoindirubin-*N*-rhamnoside β-**41a**, all glycosides were obtained as pure *Z*-configured isomers (determination by NMR, the other isomer was not visible, *E*/*Z* > 98:2). Deprotection of *E*-β-**43a**,**b** under acidic conditions gave rhamnosides *E*-β-**44a**,**b** in good yields. However, deprotection of *E*-β-**43c**–**e** failed.

**Scheme 28:**
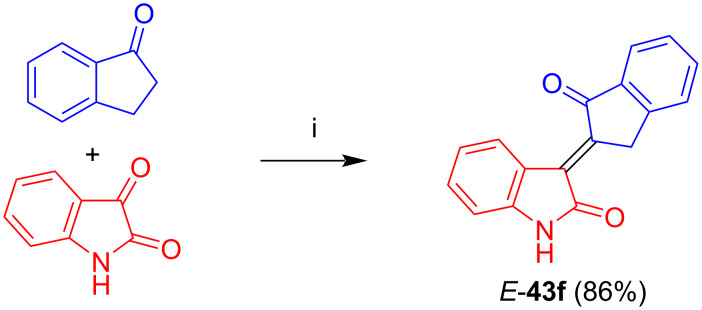
Synthesis of *E*-**43f**. Reagents and conditions: i) Na_2_CO_3_, MeOH, 20 °C, 6–24 h.

Kornienko et al. reported the synthesis and antiproliferative activity of non-glycosylated carboindirubin *E*-**43f** in 86% yield by reaction of indan-1-one (**42a**) with isatin using sodium carbonate in methanol (Scheme 28) [40]. For this compound, only moderate or no antiproliferative activities were observed against various cancer cell lines.

#### 3-Alkylideneoxindole-*N*-glycososides

**Scheme 29:**
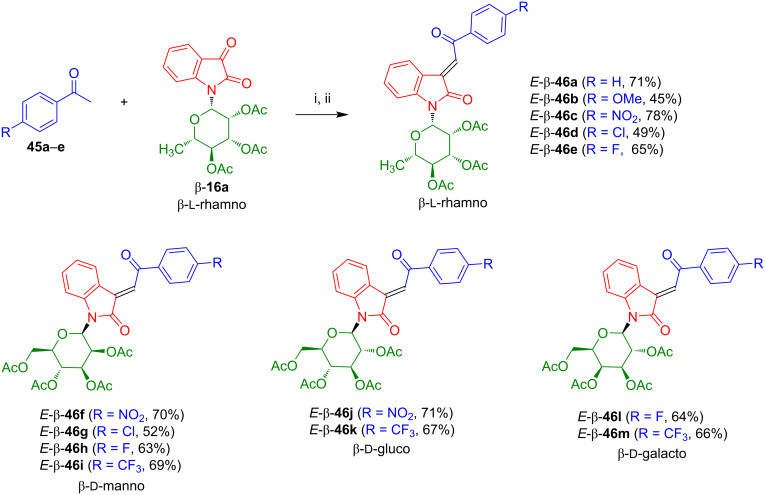
Synthesis of **46a**–**m**. Reagents and conditions: i) NEt_3_ (1 equiv), EtOH, 20 °C, 6–10 h; ii) MsCl, NEt_3_, DMAP, CH_2_Cl_2_, 0–20 °C, 2 h, 20 °C, 6–8 h.

3-Alkylideneoxindoles are of considerable pharmacological relevance and occur in a variety of clinically used drugs and natural products [42–45]. The reaction of isatin-*N*-rhamnoside **16a** with acetophenone (**45a**) afforded the desired 3-alkylideneoxindole-*N*-rhamnoside *E*-β-**46a** in good yield as an orange solid (Scheme 29) [46]. In the first step, a base-mediated aldol reaction was carried out. The corresponding alcohol was directly used as the crude product and treated with tosyl chloride, NEt_3_, and DMAP (stirring for 2 h at 20 °C) to give the corresponding tosylate which was further stirred for 6 h at 20 °C to give *E*-β-**46a**. Likewise, rhamnosides *E*-β-**46b**–**e**, mannosides *E*-β-**46f**–**i**, glucosides *E*-β-**46j**,**k**, and galactosides *E*-β-**46l**,**m** were prepared. 3-Alkylideneoxindole-*N*-glycososides **46** can be regarded as analogues of indirubin-*N*-glycosides completely missing the nitrogen atom of the indoxyl moiety.

It was demonstrated that 3-alkylideneoxindole-*N*-glycosides **46** show a significant antitumor activity against melanoma human cell lines SK-Mel-147 and A-375 [46]. As mentioned above, the death ligand TRAIL is a promising strategy for cancer treatment. Unfortunately, melanoma cells show both preexisting and inducible TRAIL resistance. Similarly, as in case of the activity of selenoindirubin-*N*-glycosides **38** against lung cell carcinoma (Scheme 23), inhibition of melanoma cell proliferation and loss of cell viability were significantly increased by the presence of the death ligand TRAIL. The antitumor effects were related to the inhibition of the survival pathway of c-Jun and JNK2 (Jun N-terminal kinase).

In an additional study, it was shown that treatment with derivative *E*-β-**46b** results in an increase of melanoma cell sensitivity for death ligands and allows to overcome resistance against TRAIL and CD95 agonists [47]. A detailed investigation of apoptotic signaling pathways revealed that TRAIL resulted, based on a negative feed-back loop, in a rapid downregulation of both agonistic TRAIL receptors DR4 and DR5. However, the presence of compound *E*-β-**46b** resulted in a compensation of this negative feed-back loop by TRAIL and an upregulation of both receptors. In addition, a loss of mitochondrial membrane potential and release of cytochrome c was observed which gave rise to an activation of intrinsic apoptosis pathways. The mitochondrial response seems to be related to an upregulation of Bax and Bad and to downregulation of Mcl-1. In fact, the presence of compound *E*-β-**46b** in combination with TRAIL was also able to overcome apoptosis resistance, due to overexpression of ectopic Bcl-2.

The proapoptotic effects of compound *E*-β-**46e** in melanoma cells were investigated in more detail. In fact, the downstream signaling pathways seem to be based on the production of reactive oxygen species [48]. Like *E*-β-**46b**, the presence of derivative *E*-β-**46e** appears to control apoptosis in melanoma cells which is (again) increased by the presence of TRAIL and results in complete loss of cell viability. As in case of compound *E*-β-**46b**, the signaling cascade was investigated also for derivative *E*-β-**46e**. Important biochemical steps were again activation of caspase-3, downregulation of XIAP, upregulation of p53 and TRAIL receptor 2, STAT-3 dephosphorylation, and loss of mitochondrial membrane potential. The most important biomolecular step, however, was the early production (already after one hour) of reactive oxygen species (ROS). This was demonstrated by pretreatment with antioxidants which completely stopped induction of apoptosis and of the loss of cell viability and resulted in disappearance of the signaling effects mentioned above. In fact, ROS were shown to be upstream of all proapoptotic signaling and to play a decisive role in apoptosis regulation which might lead to new strategies for the therapy of melanoma.

As mentioned above, cutaneous squamous cell carcinoma (CSCC) is the second most common non-melanoma skin cancer. In collaboration with Jürgen Eberle and his team, the activity of *E*-β-**46b**, *E*-β-**46e**, and *E*-β-**46k** in four CSCC cell lines were investigated [49]. High activities were observed for SCL-I, SCL-II, SCC-12, and SCC-13 cell lines with up to 80% reduction of cell proliferation, 60% reduction of cell viability and 30% induced apoptosis at a 10 μM level. Like in case of the studies mentioned above, apoptosis was further increased in combination with TRAIL. Induction of reactive oxygen species were again mandatory for these effects to be observed. In fact, apoptosis was entirely cancelled by antioxidative pretreatment and cancer cell viability and proliferation were fully regenerated. Complete activation of cascades of caspases-3, -4, -6, -7, -8, and -9, loss of mitochondrial membrane potential, activation of proapoptotic PKCδ (protein kinase C delta), and inhibition of STAT3 (signal transducer and activator of transcription 3) were observed as biochemical pathways, along with downregulation of antiapoptotic XIAP (X-linked inhibitor of apoptosis protein) and surviving as well as upregulation of the proapoptotic Bcl-2 protein Puma and the cell cycle inhibitor p21. It is worth to be noted that all these activation steps were stopped by the presence of antioxidants which indicates that ROS act as key regulators of the antitumor effects of 3-alkylideneoxindole-*N*-glycosides **48**.

In the last decades, we have witnessed an increasing incidence of cutaneous T-cell lymphoma (CTCL) which is, in fact, a dangerous form of non-Hodgkin’s lymphomas (NHL) and there is a need for innovative therapies. In collaboration with Jürgen Eberle and his team it was shown that the action of 3-alkylideneoxindole-*N*-glycoside *E*-β-**46d** results in a strong decrease of CTCL proliferation and viability and in induction of apoptosis [50]. The presence of this compound results in downregulation of the caspase antagonistic proteins c-FLIP and XIAP via caspase-8 and caspase-3 in the (extrinsic) apoptosis cascade. Similarly as discussed above for the activity of 3-alkylideneoxindole-*N*-glycosides against melanoma and CSCC, a strong increase of the concentration of reactive oxygen species (ROS) as a rapid effect is observed in response of the treatment with *E*-β-**46d**.

#### 3-(5-Oxo-2-thioxoimidazolidin-4-ylidene)indolin-2-one-*N*-glycosides

**Scheme 30:**
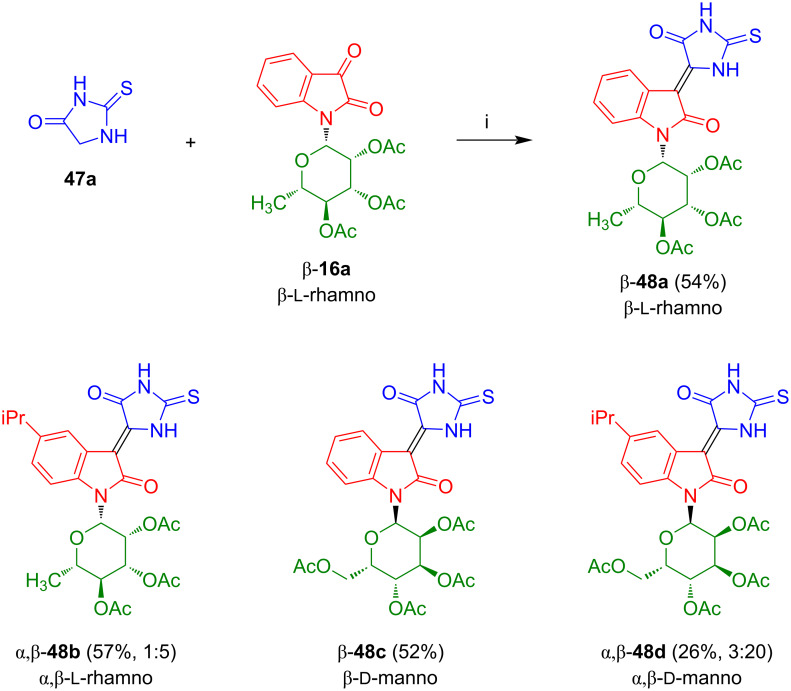
Synthesis of **48a**–**d**. Reagents and conditions: i) AcOH/Ac_2_O, NaOAc, 60 °C, 3–4 h.

The reaction of isatin-*N*-rhamnoside β-**16a** with thiohydantoin (**47a**), carried out in our group, afforded red colored β- and *Z*-configured 3-(5-oxo-2-thioxoimidazolidin-4-ylidene)indolin-2-one-*N*-rhamnoside β-**48a** in 54% yield (Scheme 30) [51]. Likewise, rhamnoside β-**48b** and mannosides β-**48c** and β-**48d** were prepared. However, all attempts to achieve a deprotection failed. Compounds **48a**–**d** were investigated with regard to their antiproliferative activities against lung carcinoma cells (H157) and human corneal epithelial cells (HCEC). All compounds showed a moderate and selective activity against H157 with 60% inhibition for compound **48b** at a concentration of 100 µM. No activity was observed against HCEC. The activities were higher as compared to those observed for selenoindirubin-*N*-glycosides β-**38a**–**h** employed in the same assay.

**Scheme 31:**
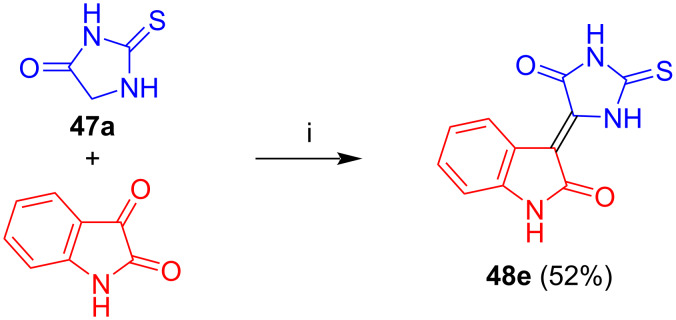
Synthesis of **48e**. Reagents and conditions: i) NaOAc, AcOH, 110 °C, 24 h.

Kornienko et al. reported the reaction of isatin with thiohydantoin (**47a**) to give condensation product **48e** (Scheme 31) [40]. This compound only showed moderate or low antiproliferative activity against various cancer cell lines.

#### 3-(2-Acetamido-4-oxo-4*,*5-dihydrothiazol-5-ylidene)indolin-2-one-*N*-glycosides

**Scheme 32:**
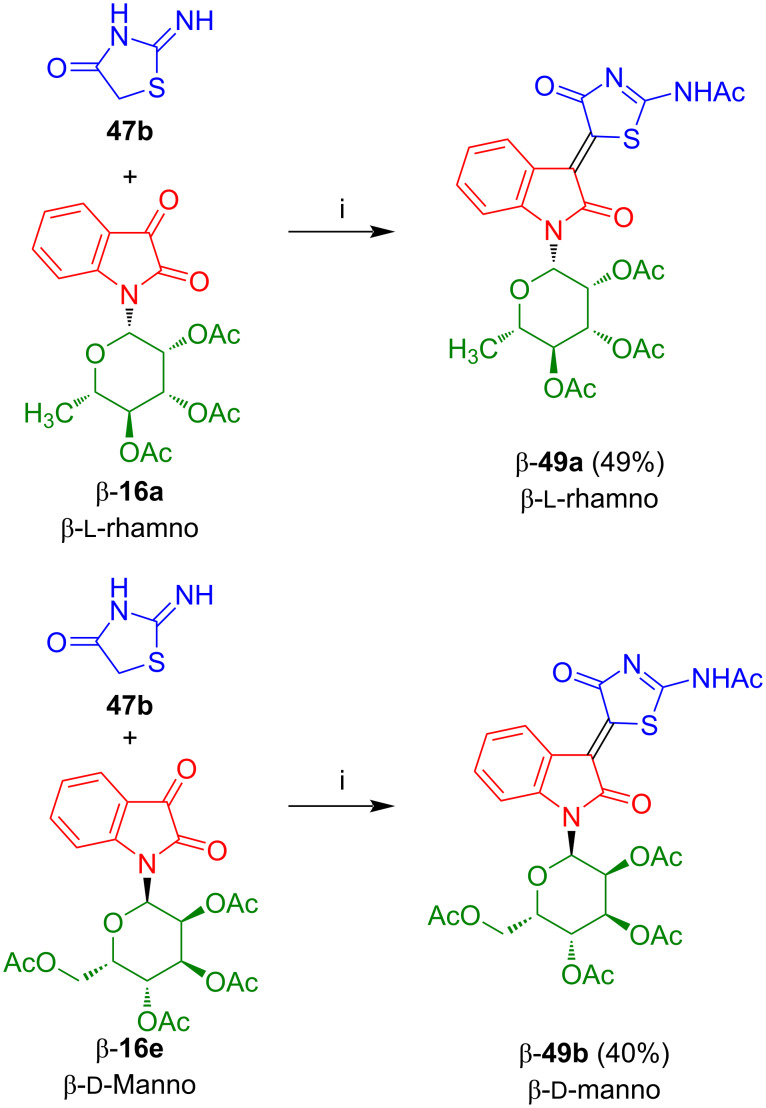
Synthesis of β-**49a**,**b**. Reagents and conditions: i) AcOH/Ac_2_O, NaOAc, 60 °C, 3–4 h.

The reaction of isatin-*N*-glycosides β-**16a** and β-**16e** with pseudo-thiohydantoin (**47b**) afforded β- and *Z*-configured 3-(2-acetamido-4-oxo-4*,*5-dihydrothiazol-5-ylidene)indolin-2-one-*N*-glycosides β-**49a** and β-**49b**, respectively (Scheme 32) [51]. Again, a deprotection was not possible under various conditions. Compounds **49a**,**b** showed a moderate and selective activity against lung cancer cells H157 [52].

#### 3-(4-Oxo-4,5-dihydrothiazol-5-ylidene)indolin-2-one-*N*-glycosides

**Scheme 33:**
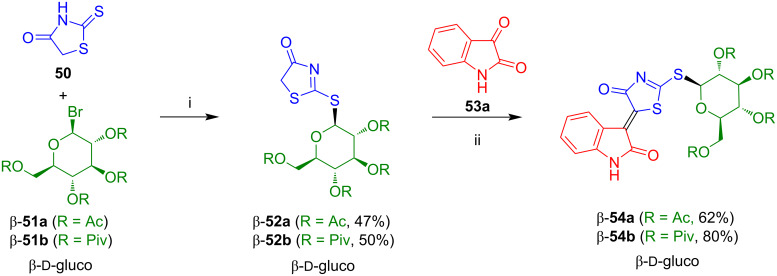
Synthesis of β-**54a**,**b**. Reagents and conditions: i) 1) NaH, DMF, 0 °C, 15 min, 2) β-**51a**,**b**, 20 °C, 3 h; ii) EtOH or MeOH/THF, cat. piperidine, 20 °C, 1–2 h.

The reaction of rhodanine (**50**) with glucosyl bromide β-**51a** afforded the acetyl-protected thiazolone-*S*-glucoside β-**52a** in 47% yield (Scheme 33) [51]. Condensation of the latter with isatin (**53a**) afforded 3-(4-oxo-4,5-dihydrothiazol-5-ylidene)indolin-2-one-*S*-glucoside β-**54a** in 62% yield and with very good *Z*-selectivity. Employment of the pivaloyl instead of the acetyl protective group resulted in an improved yield for both steps to give *S*-glucoside β-**54b**. All attempts to deprotect compounds **54a**,**b** failed. Compound **54a** shows a moderate and selective activity against lung cancer cells H157 [52].

**Scheme 34:**
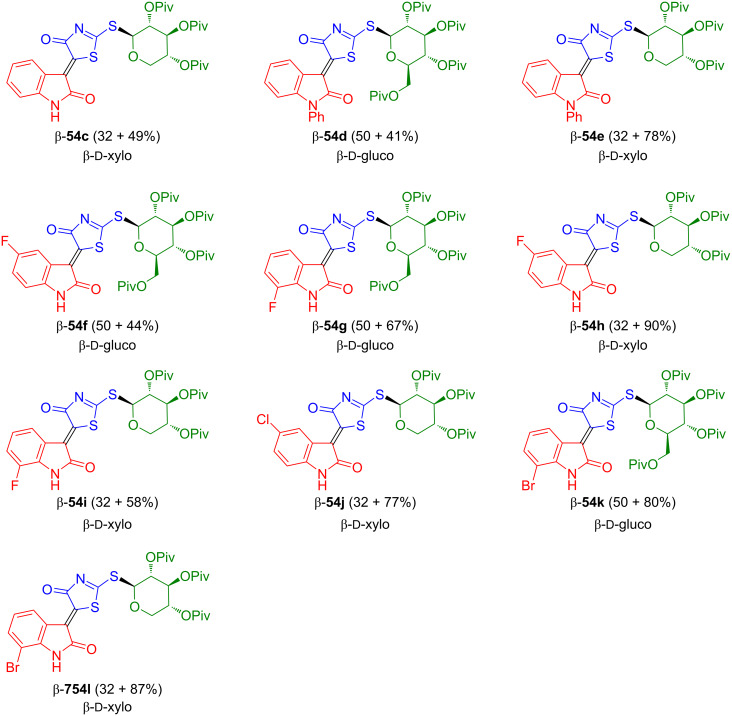
Synthesis of **54c**–**l**. The yields refer to the yields of the first and second condensation step for each compound.

The reaction of **50** with glucosyl and xylosyl bromides β-**51a**,**b** and isatins **53a**,**b** afforded pivaloyl-protected 3-(4-oxo-4,5-dihydrothiazol-5-ylidene)indolin-2-one-*S*-glycosides β-**54c**–**l** which, unfortunately, again could not be deprotected (Scheme 34) [51].

### Isoindigo-*N*-glycosides (yellow sugars)

#### Isoindigo-*N*-glycosides

**Scheme 35:**
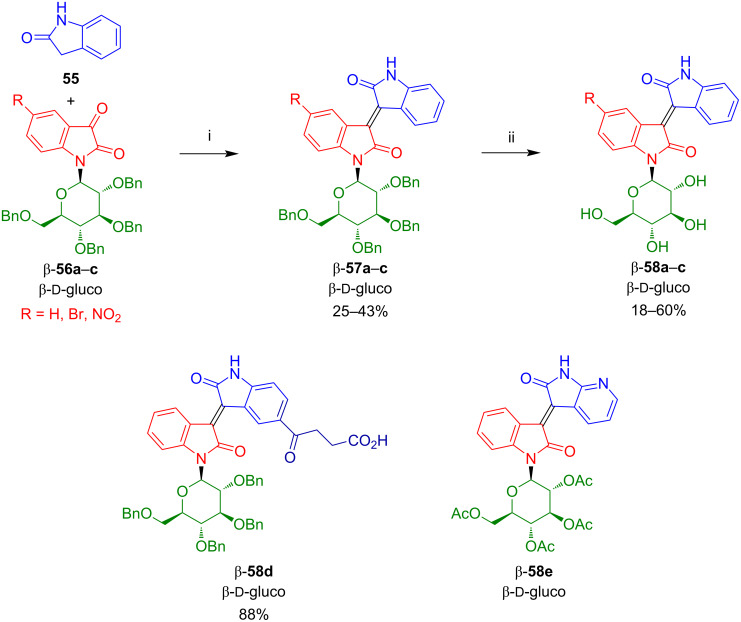
Synthesis of **57a**–**c** and **58a**–**d**. Reagents and conditions: i) HCl (conc.), AcOH, reflux, 24 h; ii) 1) BBr_3_, CH_2_Cl_2_, 20 °C, 2 h, 2) H_2_O, −78 °C.

Sassatelli et al. reported the synthesis of isoindigo-*N*-glycosides β-**57a**–**c** by reaction of benzyl-protected isatin-*N*-glycosides β-**56a**–**c** with oxindole (**55**) (Scheme 35) [53–55]. Deprotection by BBr_3_ afforded β-**58a**–**c**. Isatins **56** were prepared by glycosylation of indoline, benzylation, and oxidation with CrO_3_. Although this approach allowed for the synthesis of acceptor-substituted isatin-*N*-glycosides, it proved to be tedious and the yields were low. In a similar way, acetyl-protected isoindigo-*N*-glycosides were prepared. In addition, benzyl-protected isoindigo-*N*-glycoside β-**58d**, containing a 4-oxobutanoic acid side chain at the 5'-position, was prepared by reaction of the corresponding oxindole with isatin-*N*-glycoside **56a**. The in vitro antiproliferative activities of compounds β-**57a**–**c** and β-**58a**–**d** were studied toward a panel of human solid cancer cell lines (PC 3, DLD-1, MCF-7, M4Beu, A549, PA 1), a murine cell line (L929), and a human fibroblast primary culture. The most potent compound was β-**58d**.

Bouchikhi et al. reported the synthesis, kinase inhibitory potencies, and in vitro antiproliferative activities of acetylated 7'-azaisoindigo-*N*-glycosides, such as β-**58d** (Scheme 35) [56–57]. The presence of an additional nitrogen atom in the isoindigo moiety proved to be important for an improved antitumor activity. In vitro antiproliferative activities of these compounds were tested against the human buccal carcinoma cell line (KB) and human myeloid leukaemia cell lines (K562, HL60). While isoindigo-*N*-glycosides did not show cytotoxicity, two azaisoindigo-*N*-glycosides exhibited significant antiproliferative activities with cell proliferation inhibition of all the cell lines tested in a 75–80% range. The best activity was observed, in fact, for compound β-**58d**.

#### Isoindigo-*N,N‘*-diglycosides

**Scheme 36:**
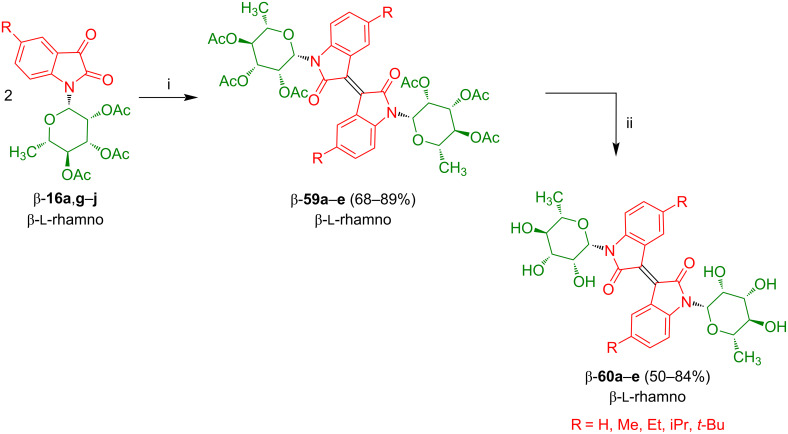
Synthesis of **59a**–**e** and **60a**–**e**. Reagents and conditions: i) P(NEt_2_)_3_ (1.1 equiv), CH_2_Cl_2_, −78 °C to 25 °C; ii) NaOMe, MeOH (abs).

In our group, the synthesis of *N,N’*-diglycosylated isoindigos was studied. In contrast to isatin-*N*-glycosides, oxindole-*N*-glycosides are not readily available. Therefore, reductive dimerization of isatin-*N*-glycosides was considered as a strategy for the synthesis of the target compounds. Isatin-*N*-rhamnosides β-**16g**–**j** were prepared from the corresponding anilines as exemplified above for the synthesis of β-**16a**. Treatment of β-**16a**,**g**–**j** with tris(diethylamino)phosphine resulted in formation of dimers β-**59a**–**e** in good yields (Scheme 36) [58]. Deprotection afforded the desired isoindigo-*N,N‘*-dirhamnosides β-**60a**–**e** as orange to brown solids.

**Scheme 37:**
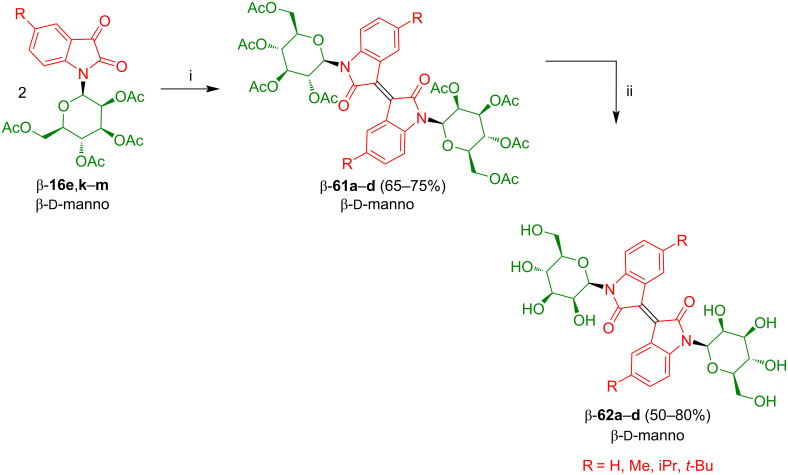
Synthesis of **61a**–**d** and **62a**–**d**. Reagents and conditions: i) P(NEt_2_)_3_ (1.1 equiv), CH_2_Cl_2_, −78 °C to 25 °C; ii) NaOMe, MeOH (abs).

Similarly to the synthesis of rhamnosides **59**, the dimerization of isatin-*N*-mannosides β-**16e** and β-**16k–m** afforded isoindigo-*N,N‘*-mannosides β-**61a**–**d** which were subsequently deprotected to give products β-**62a**–**d** in good yields (Scheme 37) [58]. The dimerization failed for isatin-*N*-glycosides derived from β-ᴅ-galactose, β-ᴅ-glucose, and β-ᴅ-xylose which might be due to their 1,2-*trans* configuration. An identical relative configuration (1,2-*trans*; 1,3-*cis*) is observed for the first three carbon atoms of β-ᴅ-galactose, β-ᴅ-glucose, and β-ᴅ-xylose. In contrast, for β-ᴅ-mannose and β-ʟ-rhamnose, the first three C-atoms possess a different relative configuration (1,2-*cis*; 1,3-*cis*). This difference cannot be explained at the moment. A possible explanation is based on the assumption that, during dimerization, the acetate group of the 1,2-*trans*-configured sugars interacts with the carbenoid intermediate of the isatin and, thus, exerts an unfavorable influence.

#### Oxoisoindigo-*N*-glycosides

**Scheme 38:**
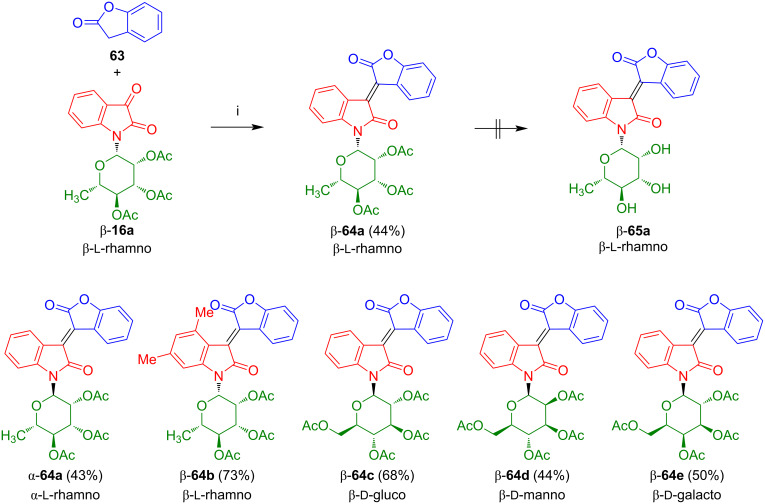
Synthesis of β-**64a**–**e** and α-**64a**. Reagents and conditions: i) AcOH, Ac_2_O, NaOAc, 90 °C, 6 h.

The reaction of isatin-*N*-rhamnoside β-**16a** with 2-coumaranone (**63**), carried out in our group, afforded the acetyl-protected oxoisoindigo-*N*-rhamnoside β-**64a** as an orange to red solid in 44% yield (Scheme 38) [59]. Unfortunately, all attempts to achieve a deprotection failed, due to cleavage of the lactone moiety. Starting with α-configured isatin-*N*-rhamnoside α-**16a**, anomerically pure α-**64a** could be prepared in 43% yield. Likewise, *N*-rhamnoside β-**64b**, *N*-glucoside β-**64c**, *N*-mannoside β-**64d,** and *N*-galactoside β-**64e** were prepared from the corresponding isatin-*N*-glycosides β-**16b**–**e** [60].

**Scheme 39:**
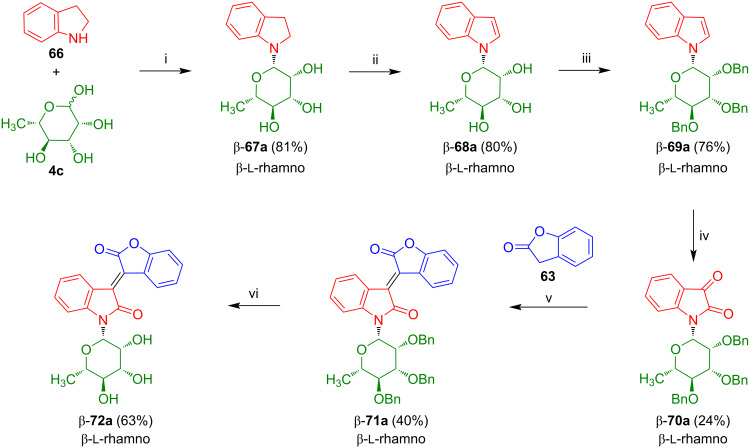
Synthesis of β-**72a**. Reagents and conditions: i) **66**, EtOH, 20 °C, 12 h; ii) DDQ, dioxane, 20 °C, 12 h; iii) NaH, BnBr, DMF, 0 °C to 4 °C, 12 h; iv) CrO_3_, acetone, AcOH, H_2_O, 20 °C, 1.5 h; v) AcOH, Ac_2_O, NaOAc, 90 °C, 2 h; vi) BBr_3_, CH_2_Cl_2_, −78 °C, 2 h.

As a consequence, we decided to employ benzyl-protected isatin-*N*-rhamnoside β-**70** which was prepared in analogy to the method of Sassatelli reported for related isatin-*N*-glycosides [53–55]. The reaction of rhamnose with indoline (**66**) afforded β-**67a** which was transformed to indol-*N*-rhamnoside β-**68a** (Scheme 39) [59]. Benzylation and subsequent oxidation gave isatin-*N*-rhamnoside β-**70a**, albeit, in low yield. Condensation with 2-coumaranone (**63**) afforded the benzylated oxoisoindigo-*N*-rhamnoside β-**71a** which was successfully deprotected to give the desired product β-**72a**. It is worth to be noted that isatin-*N*-rhamnoside β-**70a** is not available by cyclization of the corresponding benzyl-protected aniline-*N*-rhamnoside with oxalyl chloride or by deacetylation of β-**16a** and subsequent benzylation.

**Scheme 40:**
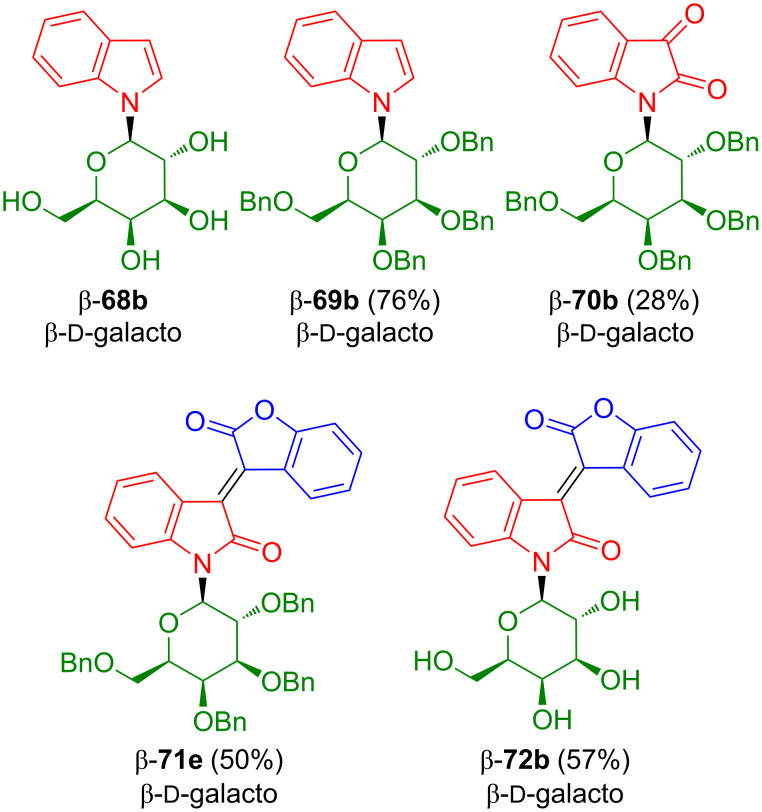
Synthesis of β-**72b**.

Benzylation of indol-*N*-galactoside β-**68b** afforded β-**69b** which was oxidized to β-**70b** (Scheme 40) [60]. Condensation of the latter with 2-coumaranone (**63**) afforded oxoisoindigo-*N*-galactoside β-**71b** which was deprotected to give β-**72b**. The yields were comparable to those obtained for the corresponding rhamnosides.

#### Thioisoindigo-*N*-glycosides

**Scheme 41:**
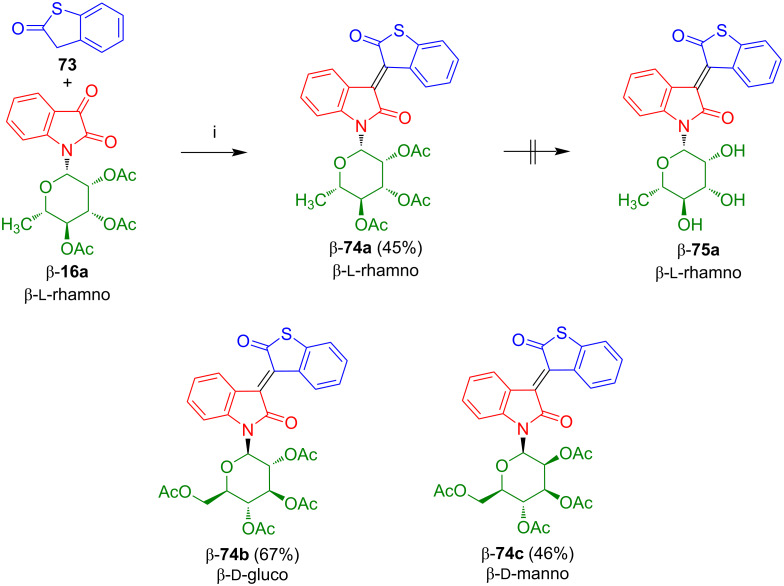
Synthesis of β-**74a**–**c**. Reagents and conditions: i) AcOH, Ac_2_O, NaOAc, 130 °C, 2 d.

The condensation of isatin-*N*-rhamnoside β-**16a** with 2,3-dihydrobenzo[*b*]thiophen-2-one (**73**), available in two steps from benzo[*b*]thiophene [61], afforded thioisoindigo-*N*-rhamnoside β-**74a** in 45% yield (Scheme 41) [60]. Like in the case of β-**64a**, deprotection proved to be unsuccessful, due to cleavage of the lactone moiety. Starting from the respective isatin-*N*-glycosides, *N*-glucoside β-**74b** and *N*-mannoside β-**74c** were prepared.

#### Carboisoindigo-*N*-glycosides

**Scheme 42:**
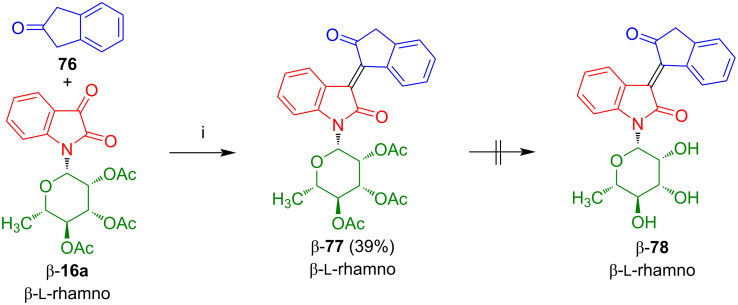
Synthesis of β-**77**. Reagents and conditions: i) 1) NEt_3_, EtOH, 20 °C, 12 h, 2) DMAP, NEt_3_, MsCl, 0 °C to 20 °C, 20 h.

The condensation of isatin-*N*-rhamnoside β-**16a** with indan-2-one (**76**) afforded carboisoindigo-*N*-rhamnoside β-**77** in 39% yield (Scheme 42) [60]. Despite the absence of a lactone moiety, deprotection proved to be unsuccessful, due to decomposition.

### Miscelleaneous

#### 6*H*-Indolo-[2,3-*b*]quinoxaline-*N*-glycosides

**Scheme 43:**
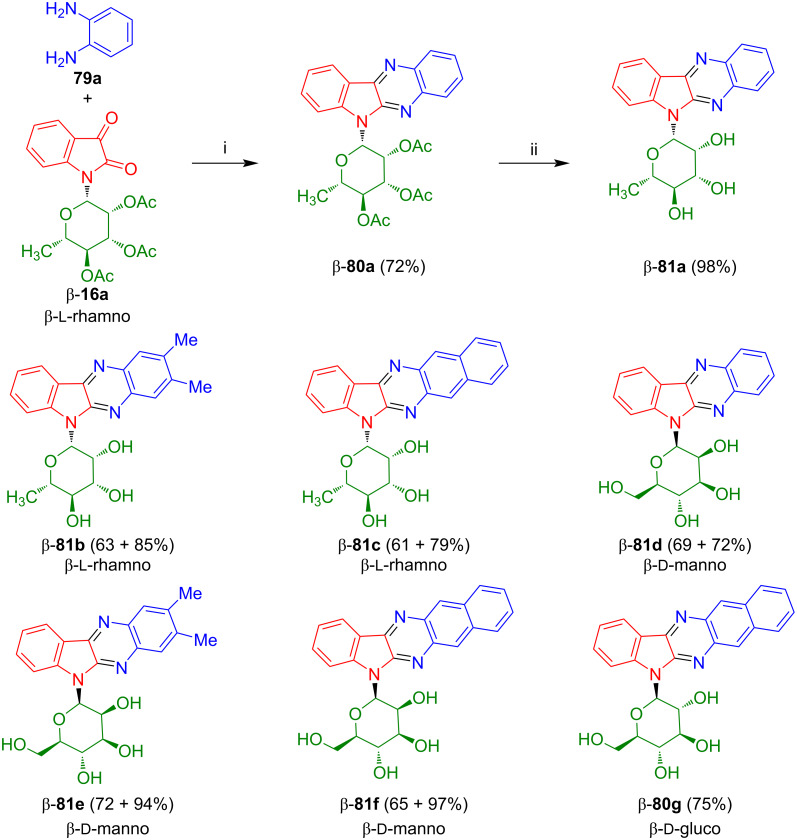
Synthesis of β-**81a**–**f** and β-**80g**. Reagents and conditions: i) AcOH, 80 °C, 1–3 h; ii) benzene, PTSA, 80 °C, 1–3 h; iii) NaOMe, MeOH, 7 h, 20 °C. The yields of **81b**–**f** refer to the yields of the cyclocondensation and the deprotection step for each compound.

The cyclization of isatin-*N*-rhamnoside β-**16a** with 1,2-diaminobenzene (**79a**) afforded 6*H*-indolo-[2,3-*b*]quinoxaline-*N*-glycoside β-**80a** in 72% yield (Scheme 43) [62]. The first step, the condensation of the amine with the (more reactive) keto group of β-1**6a**, was carried out in glacial acetic acid (80 °C). Subsequent treatment with with *p*-toluenesulfonic acid (PTSA) resulted in cyclization via the lactam to give β-**80a** which upon deprotection with NaOMe afforded β-**81a** in 98% yield. Likewise, rhamnosides β-**81b**,**c** mannosides β-**81d**–**f**, and glucoside **β-80g** were obtained in good yields. Some products exhibited weak cytotoxic activity against human ceratinocytes (HaCaT). However, in contrast to many indirubin-*N*-glycosides and their analogues described above, glycosides **81** were inactive against cancer and melanoma cell lines.

#### 3,3-Diaryloxindole-*N*-glycosides

**Scheme 44:**
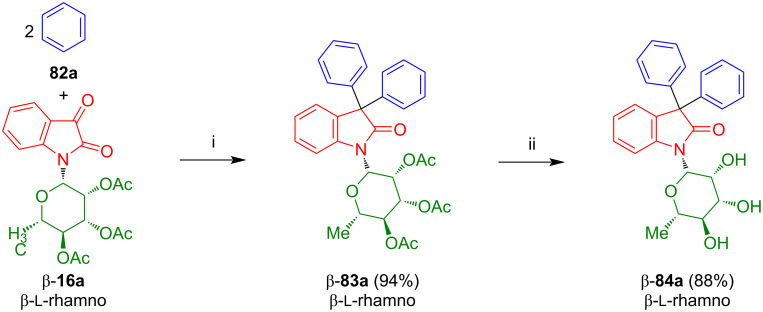
Synthesis of **84a**. Reagents and conditions: i) benzene, AlCl_3_, 20 °C, 10 min; ii) MeOH, NaOMe, 12 h, 20 °C.

The Friedel–Crafts acylation of isatin-*N*-rhamnoside β-**16a** with benzene (**82a**) afforded 3,3-diphenyloxindole-*N*-rhamnoside β-**83a** in 94% yield (Scheme 44) [63]. Deprotection of the latter afforded β-**84a** in 88% yield.

**Scheme 45:**
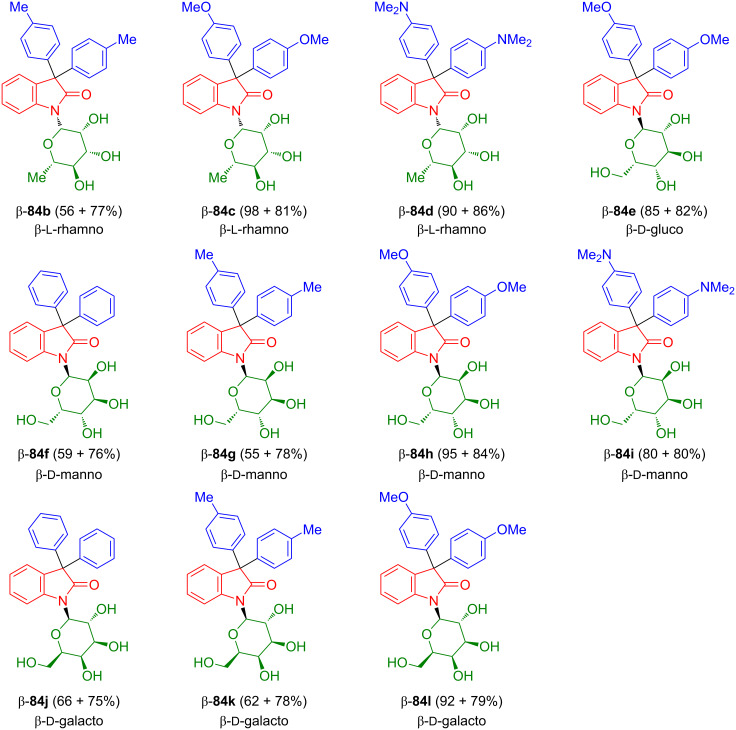
Synthesis of **84b**–**l**. The yields refer to the yields of the condensation and the deprotection step for each compound.

Friedel–Crafts acylation of isatin-*N*-glycosides β-**16c**–**e** with benzene, toluene, anisole, and *N,N*-dimethylaniline and subsequent deprotection afforded 3,3-diaryloxindole-*N*-glycosides β-**84b**–**l** (Scheme 45) [63]. Some of the products showed antiproliferative activity against malignant cutaneous melanoma cells HT-144 (ATCC HTB-63) and lung carcinoma (H157) cell line (ATCC CRL-5802).

## Conclusion

Since the 1990s, we are witnessing a renaissance of the chemistry of the old pigment dyes indigo, indirubin, and isoindigo, due to their anticancer activity which dates back to early observations made in the context of ethno-pharmacy. The present article provides an account on the synthesis and cancerostatic activity of *N*-glycosides of indigo, indirubin, and isoindigo which can be, in fact, regarded as blue, red, and yellow sugars. While specific non-glycosylated indirubin derivatives, such as indirubin-3’-monoxime, already show a good activity against various human cancer cell lines, the presence of a carbohydrate unit attached to the amide-type nitrogen atom of the indirubin moiety results in a dramatic increase of the activity. This might be due to an improved bioavailability by an improved water solubility or ability to pass the cell membrane or by an improved recognition of the drug in the active site of the receptor. In contrast to indirubin-*N*-glycosides, indigo-*N*-glycosides are relatively unstable and not highly active against cancer, except for the natural products akashins A–C. The best activity, especially against melanoma cells, was observed for thioindigo-*N*-glycosides. The activity is improved by the action of cold plasma. 3-Alkylideneoxindole-*N*-glycososides, structurally related to indirubin-*N*-glycosides, trigger apoptosis in melanoma cells, which is enhanced by the combination with TRAIL. In conclusion, indirubin-*N*-glycosides and their analogues exhibit the highest anticancer activity observed so far for indirubin derivatives. Future studies are directed to a modification of the isatin moiety of the products for which improved syntheses have been recently developed.

## Data Availability

Data sharing is not applicable as no new data was generated or analyzed in this study.
